# EP300 promotes bladder cancer cell migration through SNAI2

**DOI:** 10.1371/journal.pone.0347209

**Published:** 2026-06-08

**Authors:** Qing Zhang, Chongjie He, Yingzhou Hong, Tao Huang, Shuangsheng Deng, Cheng Peng, Haifeng Wang, Chunming Guo

**Affiliations:** 1 Yunnan Key Laboratory of Cell Metabolism and Disease, Center for Life Sciences, School of Life Sciences, Yunnan University, Kunming, China; 2 Department of Urology, The Second Affiliated Hospital of Kunming Medical University, Kunming, China; “INSERM”, FRANCE

## Abstract

EP300, a frequently mutated transcriptional coactivator in bladder cancer, has been demonstrated to positively correlate with malignancy in various cancers. Highly aggressive bladder cancers are often associated with muscle-invasive progression, a process closely linked to the epithelial-mesenchymal transition (EMT) capability of bladder cancer. Here, we performed immunohistochemical staining on bladder cancer tissues and adjacent normal tissues obtained from radical cystectomy to validate the positive association between EP300 and bladder cancer malignancy. We observed that A485, a small-molecule inhibitor of EP300, significantly inhibited cell migration in multiple bladder cancer cell lines (SW780, T24, RT4 and 5637) both in scratch wound healing and Transwell assays. Additionally, take the advantage of organoid-3D culture system, A485 disrupted the formation of inter-organoids connecting tubular structures in T24 cell line, while A485 also disrupted the organoid migration from matrix out-toward the non-matrix region both in SW780 and 5637 cell lines. In *in vivo* experiment, A485 showed a trend toward inhibiting BBN-induced bladder cancer invasion in mice. Bulk RNAseq analysis revealed that A485 commonly downregulated SNAI2-related signaling pathways across multiple bladder cancer cell lines. Furthermore, western blotting confirmed that A485 significantly reduced the global level of acetylation of histone H3 at lysine 27 (H3K27ac) as well as SNAI2 expression in T24 and SW780 cell lines. Indeed, overexpression of SNAI2 enhanced the migratory capacity of bladder cancer cells via Transwell assay. Collectively, our findings demonstrate that EP300 promoted bladder cancer cell migration via up-regulating SNAI2, targeting EP300 could be a potential therapeutic strategy to inhibit the process of bladder cancer invasion.

## Introduction

Bladder cancer, particularly muscle-invasive bladder cancer (MIBC), remains a significant clinical challenge due to its high-grade metastatic character, and resistance to conventional therapies [1–4]. Worldwide cancer genomics analysis raised a hypothesis that epigenetic reprogramming may play a critical role in bladder cancer tumorigenesis and progression, due to multiple epigenetic genes frequently mutated in bladder cancer genome [5,6]. Among these, EP300, a histone acetyltransferase (HAT) and transcriptional coactivator, has emerged as a key player in modulating oncogenic pathways in many cancer types [7–9].

*EP300* encodes a multifunctional protein (EP300) which regulates acetylation modification upon protein lysine residue including both histone and non-histone protein, thereby influencing either the accessibility of chromatin structure or the activation of transcriptional factor [10,11]. While EP300 exhibits dual roles in cancer either tumor-suppressive [12] or oncogenic [7,8] in context dependently, its aberrant activity in bladder cancer is increasingly linked to malignant phenotypes [13]. For instance, genomic analyses reveal that EP300 mutations occur in approximately 15% of bladder cancers, often correlating with altered histone acetylation patterns and transcriptional programs that drive proliferation, invasion, and immune evasion [14,15]. Moreover, EP300-mediated acetylation of histone 3 lysine 27 site [16] has been implicated in enhancing chromatin accessibility at oncogenic loci, facilitating epithelial-mesenchymal transition (EMT) and metastasis in multiple cancer types [17].

EMT is a hallmark of invasive progression in most cancer types, characterized by loss of E-cadherin and upregulation of markers like Vimentin and SNAI2 [18]. Studies have suggested that EP300 might promote EMT by acetylating β-catenin, a key effector of the Wnt pathway, thereby stabilizing its nuclear localization and activating pro-migratory genes [19–21]. Furthermore, EP300 regulated inflammatory signaling pathways such as NLRP3 [22], which is upregulated in bladder cancer under inflammatory microenvironments, further promoting cell migration and invasion [23]. These findings underscored the multifaceted role of EP300 in reshaping the bladder cancer epigenome toward a metastatic phenotype.

Despite these studies, the precise mechanisms by which EP300 drives bladder cancer cell migration remain incompletely understood. In this study, human bladder cancer samples were used to verify the correlation between EP300 and malignancy degree, and the inhibition effect of EP300 small molecule inhibitor A485 was used to test the migration ability of bladder cancer cells both in two-dimensional (2D-) and three-dimensional (3D-) culture and BBN(N-butyl-N-(4-hydroxybutyl) nitrosamine)-induced spontaneous bladder cancer mice models [24,25]. Our results suggested EP300 may be a potential intervention candidate for bladder cancer infiltration.

## Materials and methods

### Human samples

The bladder cancer tissues from the patients were obtained from the Second Affiliated Hospital of Kunming Medical University, and all studies were performed according to protocols reviewed and approved by Medical Ethics Committee: FEY-BG-39–2.0/审-PJ-科-2023−246, FEY-BG-38–1.2/审-YJ-2024–114.

### Animals

The study involving animals was conducted in compliance with approved protocols (YNU20220176, YNU20230525) from the Institutional Animal Care and Use Committee (IACUC) at Yunnan University, adhering to all applicable guidelines. C57BL/6J mice (JAX#000664) were acquired from The Jackson Laboratory.

### BBN-induced bladder cancer mice model *in vivo*

Bladder cancer was induced in male mice by administration of 0.1% BBN (Sigma) via the drinking water for a duration of 20 weeks, commencing at 8–10 weeks of age. DMSO (Sigma, Cat. No. D2650) or 5 mg/kg A485 (MCE, Y-107455) were injected i.p. twice a week after two-months of BBN treatment. At the endpoint, mice were euthanized, and bladder tissues were excised, embedded, sectioned, and stained.

### Cell lines and cell culture

RT4，SW780，T24 and 5637 cells are purchased from the Cell Bank of the Chinese Academy of Sciences. Among them, RT4 (derived from bladder papillary transitional cell carcinoma) and SW780 (derived from bladder transitional cell carcinoma) exhibit low malignancy, 5637 (derived from Grade II bladder cancer) shows intermediate malignancy, while T24 (derived from metastatic bladder cell adenocarcinoma) demonstrates the highest malignancy level. SW780 and 5637 cells were cultured in RPMI 1640 medium (Gibico, C11875500BT). RT4 and T24 were cultured in McCoys 5A medium (VivaCell, C3020-0500). Both mediums were supplemented with 10% fetal bovine serum (BI, 04-001-1ACS) and 1% penicillin/streptomycin (Hyclone, SV30010). 1640 medium requires an additional 1% HEPES (VivaCell, 03–025-1B). All cultures were maintained at 37 °C in an atmosphere of 5% CO2. All inhibitor tests were treated with 10μM A485 for 24hrs. For 3D culture, 5μl complete medium containing 800 cells was mixed with 70μl matrix gel (R＆D, 3533-010-02) and transferred to a 24-well plate with a pipette tip to form a hemispherical shape. After solidifying at 37°C in cell incubator for 30 minutes, 600μl of complete medium was added in a well for continued culture.

### Antibodies

Anti-EP300 (Santa Cruz, sc-32244), anti-KRT5 (Abcam, ab53121), anti-E-cad (CST, #3195), anti-N-cad (CST, #13116), anti-Claudin-1 (CST, #13995), anti-SNAI2 (CST, #9585), anti-TWIST1 (CST, #90445), anti-ZEB1 (CST, #70512), anti-GAPDH (Engibody, AT0002), anti-H3K27ac (Abcam, ab4729), anti- Histone3 (Engibody, AT1576), Anti-rabbit IgG, HRP-linked Antibody (CST, #7074S), Anti-mouse IgG, HRP-linked Antibody (CST, #7076S).

### Hematoxylin and eosin staining

Bladder cancer fresh tissues were fixed in 4% paraformaldehyde (PFA), embedded in paraffin and sectioned at a thickness of 5 µm. The sections were de-paraffinized and stained in Hematoxylin solution for 4 minutes followed by differentiation solution and bluing solution for seconds. Then, the sections were stained in Eosin solution for 1 minutes followed by dehydration and hyalinization by 95% ethanol, anhydrous ethanol and xylene.

### Immunohistochemistry

Paraffin sections (5µm) were de-paraffinized in dewaxing solution, rehydrated through an alcohol gradient, and sections were boiled in pre-heated 0.01M citrate buffer (pH 6.0) for 20 minutes in a pressure cooker. Subsequently, sections were incubated with 1x Animal-Free Blocking Solution (CST, 15019S) for 1 hour at room temperature. Antibodies were diluted in 0.1x Animal-Free Blocking Solution and incubated overnight at 4°C. Endogenous peroxidase activity was blocked by incubation with 3% hydrogen peroxide for 15 minutes. Subsequently, the second antibody incubation and Diaminobenzidine (DAB) staining were performed according to Kit (Genesion, GK500710): Horseradish Peroxidase-linked secondary antibody was incubated at room temperature for 1 hour. DAB reagent was used as a chromogen followed by hematoxylin staining.

### Migration assay

For the scratch wound assay, 2 × 10^5^ cells/well were plated into a 6-well plate and incubated to reach confluence. Three wounds were scratched on the monolayer using a 200μl tip and washed with PBS to remove detached cells. To exclude the wound-healing effects caused by serum-induced cell proliferation, the cells were then cultured in serum-free complete medium supplemented with either DMSO or A485 (10μM). For T24 cells, the serum-free complete medium consisted of McCoys 5A and 1% penicillin/streptomycin, while for SW780 cells, it was composed of 1640 medium, 1% penicillin/streptomycin, and 1% HEPES. The same wound regions of each well were photographed at 0hr and 24hr. The closure degree of wound was calculated as follows: wound closure ratio = (D1 – D0)/D0, where D1 represents the mean width of 0h wound, D1 represents the mean distance of 24h wound.

For the Transwell assay, 1.5 × 10^5^ SW780 or 4 × 10^4^ T24 were suspended in 200μl serum-free medium and seeded into the upper chamber of Transwell 24-well plates (Corning, 3464) with 8μm pore filters. Then the lower chamber was added with 800μl complete medium (containing 10% FBS). To investigate whether A485 can inhibit the migration ability of bladder cancer cells rather than the chemotactic effect of the drug on the cells, the same concentration of DMSO or A485 was added to both the upper and lower chambers. After 24hrs, the cells attached on the upper surface of the filter membranes were cleaned and migrated cells of the lower surface were fixed with 4% PFA for 20 minutes, then stained with Giemsa Staining Solution (Beyotime, C0131) for 30 minutes. The level of migration was photographed under an optical microscope (Leica DMI6000B, Germany). The migration ratio of wound was calculated as follows: migration ratio = N1/N0, where N0 represents the mean cell number of untreated group, D1 represents the mean cell number of untreated group.

### CCK-8

Cell viability was assessed using the Cell Counting Kit-8 (BIOMIKY, MK001A) according to the manufacturers instructions. Briefly, T24 cells were seeded into 96-well plates at a density of 4 × 10³ cells per well and allowed to adhere overnight. When the cell fusion rate reaches 80%, cells were then treated with DMSO or A485 (10μM) for 2h and 24h. Following treatment, 10 µL of CCK-8 reagent was added to each well, and the plates were incubated at 37°C for 3 hours. The absorbance of each well was measured at 450 nm. All experiments were performed with five replicates and repeated independently at least three times.

### Western blotting

Protein was extracted from cells using RIPA buffer (Beyotime, P0013B) supplemented with protease inhibitors (Roche, 11697498001). The BCA Protein Assay Kit (Thermo Fisher Scientific, 23225) was used to quantify protein concentrations. Resolved by SDS–PAGE gel (Beyotime, P0469M) electrophoresis, and then transferred to PVDF membranes (Millipore, IPVH00010) followed by blocking in 5% non-fat powdered milk in TBST for 1 hour at room temperature. Primary antibodies were applied at 1:1000 dilution in TBST and incubated overnight at 4°C. Peroxidase conjugated secondary antibody (CST, 7074S, 7076S) was used to incubated for 1 hour at room temperature, and the antigen-antibody reaction was visualized by enhanced chemiluminescence assay (ECL, Thermo, 34580).

### RNA sequencing data processing

We use STAR [26] (version 2.7.2a) to align RNA-sequencing data. Human reference genome and annotation file were downloaded from GENCODE (https://ftp.ebi.ac.uk/pub/databases/gencode/Gencode_human/release_46/GRCh38.primary_assembly.genome.fa.gz,https://ftp.ebi.ac.uk/pub/databases/gencode/Gencode_human/release_46/gencode.v46.primary_assembly.annotation.gtf.gz. Then we use HTSeq [27] (version 2.0.9) to count reads within features (with parameter --minaqual 10, --stranded no, --mode union). EdgeR [28] (version 4.0.16) function exactTest was used for differential expression analysis. MDS plot was drawn with limma [29] (version 3.58.1). We use clusterProfiler [30] (version 4.10.1) to perform GO enrichment analysis.

### CUT&Tag

The CUT&Tag assay was performed using the Hyperactive Universal CUT&Tag Assay Kit for Illumina (Vazyme Biotech, TD904) following the manufacturers protocol. Briefly, 1x10⁵ cells were bound to ConA beads and incubated overnight at 4°C with a primary antibody. After washing, samples were incubated with a secondary antibody, followed by the pA/G-Tnp enzyme. Tagmentation was carried out at 37°C, and the reaction was terminated. DNA was purified using DNA Extract Beads, and libraries were amplified via PCR. Final PCR products were purified with DNA Clean Beads and prepared for sequencing.

## Results

### EP300 was positively associated with the progression of human bladder cancer

**Fig 1:**
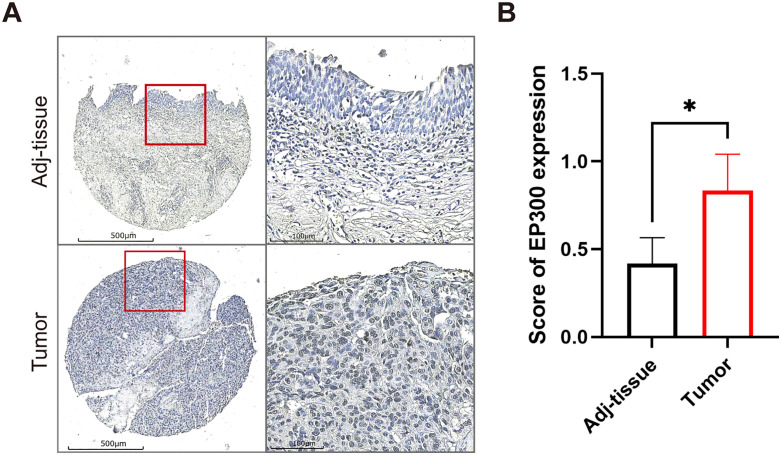
Positive correlation between EP300 and human bladder cancer malignancy. **A.** Immunohistochemical staining of EP300 in bladder cancer and tumor-adjacent tissues, scale bar, left, 500μm, right, 100μm. **B.** Immunohistochemical staining displayed EP300 expression was higher in bladder tumor compare to adjacent tissue (n = 12 pairs). *, *p < 0.05.*

To investigate the correlation between EP300 and human bladder cancer. We generated human tissue microarray chips, including 12 pairs of bladder cancer and adjacent tissues. Then we performed immunohistochemical staining for EP300 on array chips (Fig 1A). We evaluated the expression level of EP300 in each tissue sample as ranging from 0 to 4 points based on the degree of staining of nuclear EP300, and conducted statistical analysis (Fig 1B). The results showed that EP300 expression is higher in tumors than adjacent-tissues, suggesting a positive correlation between activation of EP300 and advanced tumor regions was existed. Therefore, EP300 may be related to bladder cancer invasion.

### EP300 small molecule inhibitor reduced the migration ability of bladder cancer cells

**Fig 2:**
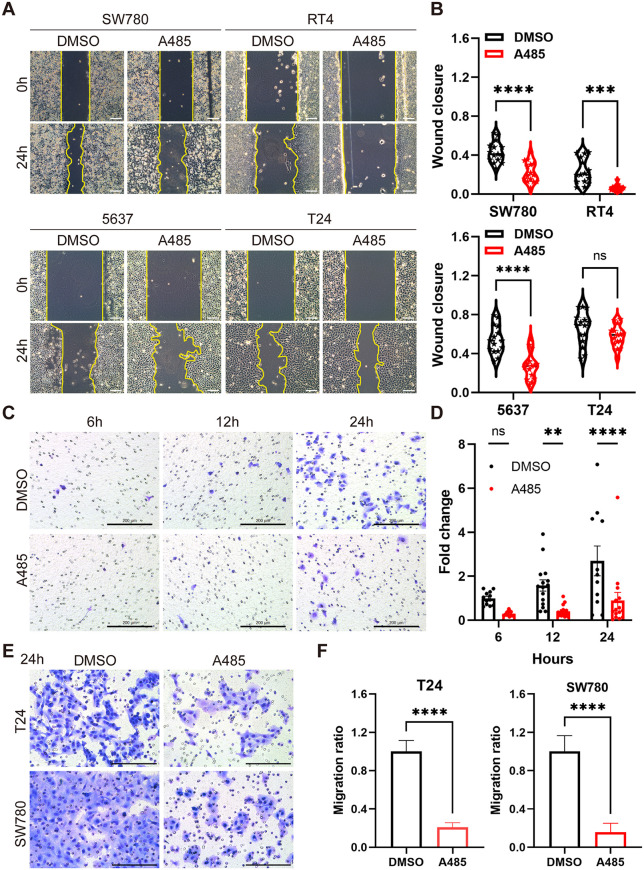
EP300 inhibition decreased human bladder cancer cell migration. **A.** The wound healing after the scratches of four types of cells with or without A485 treatment, scale bar, 100μm. **B.** Statistical analysis of wound healing assay, n ≥ 3. **C.** Transwell experiment after 6 h, 12 h and 24 h treatment with or without A485(10 μM), scale bar, 200μm. **D.** Statistical analysis of Transwell assay, n ≥ 3. **E.** Transwell experiment after 24 h treatment with or without A485(10 μM), scale bar, 200μm. **F.** Statistical analysis of Transwell assay, n ≥ 3. **, p < 0.01, ***, p < 0.001, ****, p < 0.0001.

To investigate if EP300 is required for cell migration, we employed A485, a small-molecule inhibitor which specifically targeting EP300s acetyltransferase activity. Four human bladder cancer cell lines were tested: RT4 and SW780 (derived from lower malignancy tumors) and 5637 and T24 (derived from high-malignancy tumors). In scratch assays, A485 treatment significantly attenuated wound closure capacity in three cell lines (RT4, SW780, and 5637), although T24 cell line only had a tread (Fig 2A, B). According to ATCC information, the mitotic time of T24 cells is approximately 12 hours. However, Transwell assay results showed that after A485 treatment, the migratory ability of T24 cells began to significantly decline as early as 6 hours (Fig 2C, D)。 By 24 hours, it had declined by 1.796-fold (Fig 2D) -far greater than the 0.389-fold reduction of proliferation in T24 cell observed under the same conditions as Transwell assay (S1 Fig), indicating that the inhibitory effect of A485 in the Transwell assay is majorly independent of cell proliferation. Moreover, the inhibitory effect of A485 on SW780 cell migration at 24 hours was consistent with that on T24 cells (Fig 2E, F). These results confirmed that EP300 is required for the cell migration for at least four types of human bladder cancer cell lines from various malignancy grades.

### Bladder cancer cell migration was dependent on the HAT activity of EP300

**Fig 3:**
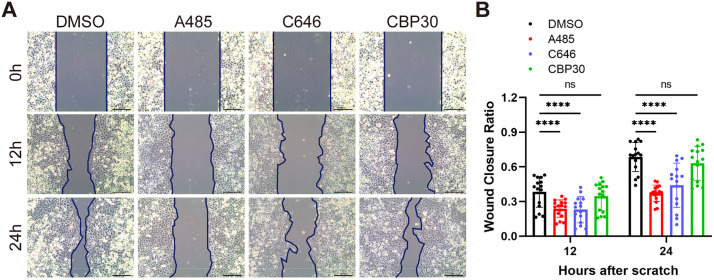
The effect of EP300 small molecule inhibitors on the migration of T24 cells. **A.** The wound healing of T24 cells after scratches under different EP300 inhibitors treatment (10 μM) at different time points, Scale bar, 200μm. **B.** Statistical analysis of wound healing assay, n = 3. ****, p < 0.0001.

Since EP300 contains multiple functional domains, including the HAT domain (the histone acetyltransferase activity center) and the BRD domain (which is involved in histone recognition) and so on, we aimed to verify that the effect of EP300 on the migration of bladder cancer cells depends on its acetyltransferase activity. In addition to the HAT inhibitor A485, we also tested the effects of another HAT inhibitor, C646, and the BRD inhibitor, CBP30, on the migration of T24 cells. The results showed that, compared to the DMSO control group, both A485 and C646 significantly inhibited wound healing in T24 cells, whereas CBP30 showed no significant difference (Fig 3A, B).. These findings indicate that the migration of T24 cells depends on the acetyltransferase activity of EP300.

### A485 inhibited the spatial connection of bladder cancer clones in 3D culture model

**Fig 4:**
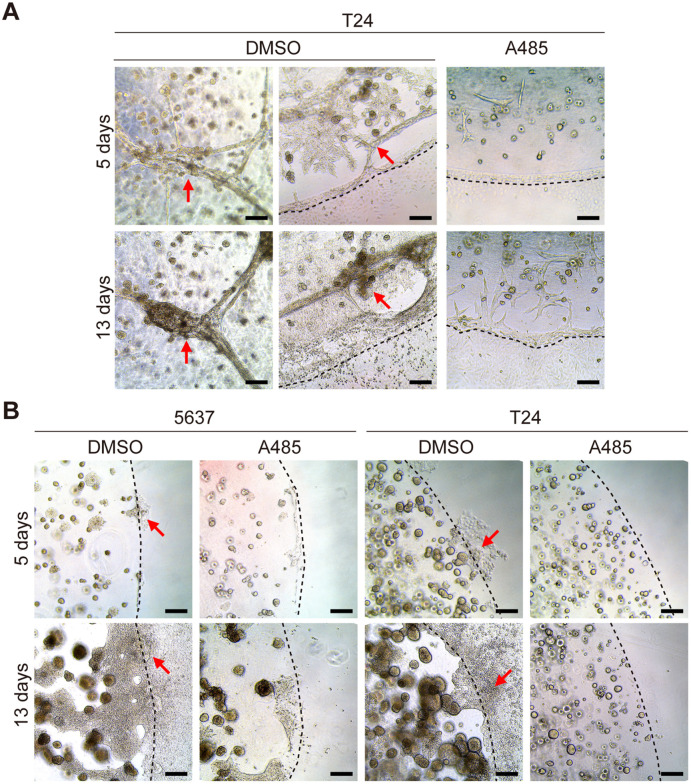
A485 inhibited migration of human bladder cancer cells in 3D culture. **A.** After treatment with A485 (10 μM), the three-dimensional tubular structure was disappeared in T24 cells. **B.** After treatment with A485 (10 μM), the invasion process was decreased in SW780 and 5637 cells. Dash line represented the edge between medium and Matrigel, arrow pointed the representative regions either protrusions or tubular and reticular networks. Scale bar, 100μm.

Compared to conventional two-dimensional 2D conditions, the three-dimensional 3D system better recapitulates the *in vivo* tumor matrix microenvironment. To further evaluate the three-dimensional migration behavior of human bladder cancer cells, we employed 3D matrigel-based model. In 3D cultures, the organoid like clones from SW780, T24, and 5637 cell lines exhibited invasive protrusions extending from the Matrigel matrix. Notably, the organoid like clones from T24 cells developed interconnected tubular and reticular networks (Fig 4A, B), and those 3D structures developed within 5 days. EP300 inhibitor A485 significantly reduced both the frequency of matrix-penetrating protrusions in SW780, T24, and 5637 cell lines and completely abolished tubular network formation in T24 cell line (Fig 4C, D). These findings provide compelling 3D evidence that EP300 is also required for the 3D level invasive capacity of bladder cancer cells across malignancy grades.

### A485 delayed invasion of BBN-induced mouse bladder cancer in trend

**Fig 5:**
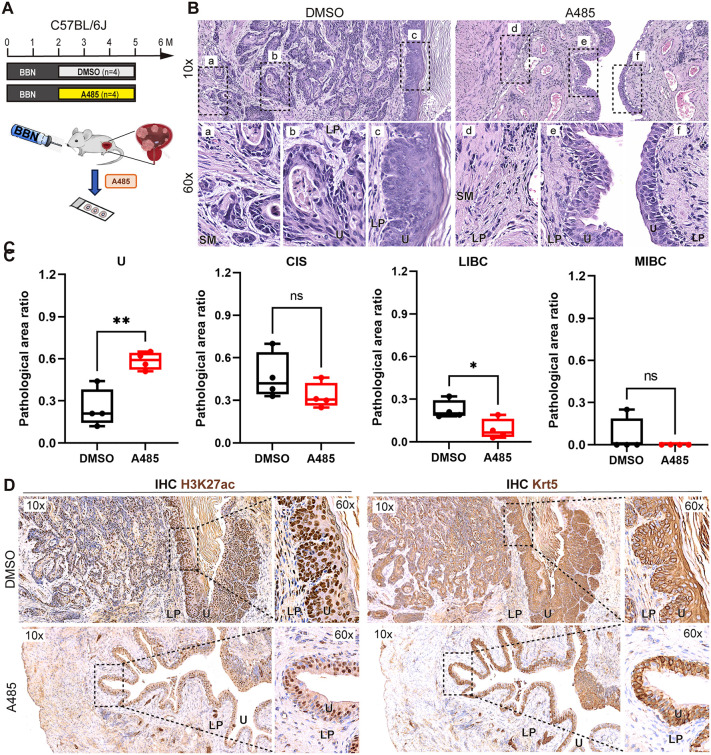
A485 inhibits BBN-induced bladder cancer invasion in mice. **A.** Schematic of A485 treatment in BBN-induced mouse model of bladder cancer **B.** H&E staining of bladders from mice treated with BBN for 5 months. **C.** Area analysis of different degrees of invasion. U, normal urothelium. CIS, carcinoma *in situ*. LIBC, lamina propria-invasive carcinoma. MIBC, muscle-invasive carcinoma. **D.** Immunohistochemistry staining for H3K27ac and Krt5. U, urothelium, LP, lamina propria, SM, smooth muscle. n = 4 mice per group. *, p < 0.05, **, p < 0.01.

To investigate the effects of A485 *in vivo*, we established a BBN-induced mouse bladder cancer model. Mice were continuously treated with BBN for five months, while DMSO or A485 treatment began two months after BBN administration and continued until the end of the experiment. Pathological evaluation of the extent of bladder cancer invasion was performed after the experiment (Fig 5A). Hematoxylin and eosin (HE) staining results showed that bladder cancer in the DMSO group invaded the muscle layer, whereas in the A485 group, invasion was limited to the lamina propria, indicating a reduced degree of invasion after A485 treatment (Fig 5B). We further quantified the proportion of different invasion types in each bladder epithelium. The results showed that after A485 treatment, the proportion of normal bladder epithelium in mice significantly increased, while the proportion of lamina propria-invasive significantly decreased (Fig 5C). Immunohistochemical staining results also demonstrated a decrease in H3K27ac levels in bladder epithelial cells (marked by KRT5) after A485 treatment (Fig 5D), suggesting that the effect of A485 on the extent of bladder cancer invasion in mice is associated with H3K27ac.

### SNAI2-related signaling pathway is down-regulated after A485 treatment

**Fig 6:**
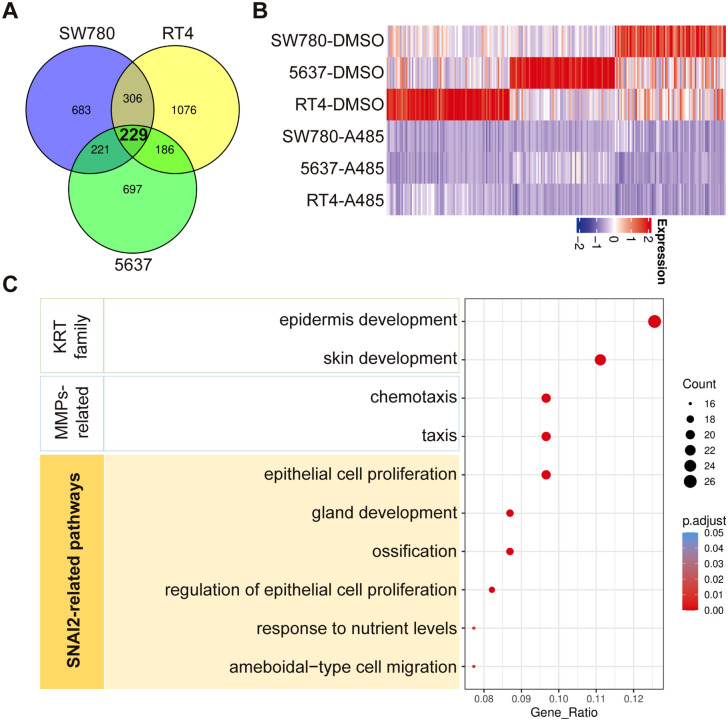
RNAseq analysis revealed EP300 may be required for cell differentiation and migration. **A.** Venn diagram analysis for DEGs from three bladder cancer cell lines. **B.** Heatmap for 229 common DEGs from three bladder cancer cell lines. **C.** GO analysis for DEGs from three bladder cancer cell lines.

EP300 is a transcriptional coactivator and maintained many aspects of cellular mechanisms. To elucidate the molecular mechanisms underlying EP300-driven migration in bladder cancer cells, we performed transcriptome sequencing (RNA-seq) analysis in three representative bladder cancer cell lines. Bioinformatic analysis identified that 229 differentiated expression genes (DEGs) were consistently downregulated genes across RT4, SW780, and 5637 cell lines (Fig 6A) upon EP300 inhibition. Heatmap visualization revealed distinct expression patterns of these genes pre- and post-treatment in all three models (Fig 6B), including differentiation marker KRT13 and cell migration associated gene SNAI2. Gene Ontology (GO) enrichment analysis demonstrated significant suppression of biological pathways critical to tumor migration, including keratin (KRT) family-mediated differentiation programs, matrix metalloproteinase (MMP)-associated extracellular matrix remodeling, and SNAI2-regulated epithelial-mesenchymal transition (EMT) (Fig 6C). Within the SNAI2-regulated EMT genes list, known SNAI2-associated genes such as ELF3, FGF1, FERMT1, FGFBP1, IGF2, IGFBP3, SPARC, SNAI2 and TP63 were found (Supplementary table 1). These findings suggested that EP300 is required both in urothelial differentiation and migration event, due to multiple signaling pathways commonly shared EMT key transcription factor SNAI2, we further tested if SNAI2 is a directly downstream of EP300 and if SNAI2 could directly reinforce the EMT event in bladder cancer cells.

### EP300 promotes bladder cancer cell migration via SNAI2

**Fig 7:**
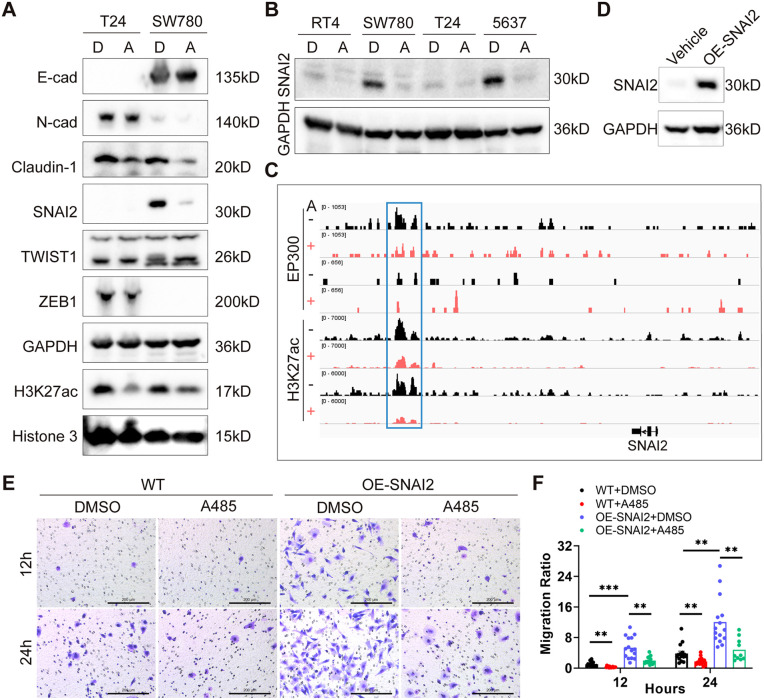
EP300 promoted bladder cancer cell migration through SNAI2. **A.** Western blot was subjected to detect EMT pathway-related protein levels in T24 and SW780 cells were decreased with or without A485 treatment (10 μM), n > 3. **B.** Western blot was subjected to detect SNAI2 protein levels in four types of bladder cancer cells with or without A485 treatment, n > 3. **C.** CUT＆Tag assay of EP300 and H3K27ac near SNAI2 gene with or without A485 treatment in SW780, n = 2, decreased regions were highlighted in blue box. **D.** Western blot was subjected to detect SNAI2 protein level in SNAI2-overexpressed T24 cells, n > 3. **E.** Transwell results of wild-type and SNAI2-overexpresed T24 cells at different times with or without A485 treatment, scale bar, left, 200μm. **F.** Statistical analysis for Transwell experiments, n = 3. **, p < 0.01, ***, p < 0.001.

As a core regulator of the epithelial-mesenchymal transition (EMT) signaling pathway, SNAI2 is upregulated during EMT progression. To validate its critical role in EP300-mediated bladder cancer cell migration, we analyzed EMT-related protein levels in T24 and SW780 cells following A485 treatment. Pharmacological inhibition of EP300 significantly reduced H3K27ac levels (confirming EP300 inactivation) and concurrently decreased SNAI2 protein expression in two cell lines (Fig 7A). This regulatory relationship between EP300 and SNAI2 was further corroborated across multiple human bladder cancer cell lines (Fig 7B). H3K27ac, the most well-known consequence of histone acetyltransferase activity of EP300, was reported to be associated with enhancer activation [31]. To verify the direct regulatory of EP300 on SNAI2, we performed CUT&Tag assays of EP300 and H3K27ac in SW780 cells. The results showed significant enrichment of EP300 and H3K27ac near SNAI2 gene region, which decreased after A485 treatment, suggesting that EP300 may regulate SNAI2 transcription through enhancer-activity (Fig 7C). To verify the function of EP300/SNAI2 axis, stable SNAI2 overexpression was carried out in SW780 cells (Fig 7D). In Transwell assay, SNAI2-overexpressed T24 cells exhibited significantly stronger migratory capacity compared to wild-type cells and reversed the inhibitory effect of A485 on cell migration. (Fig 7E, F), indicating SNAI2 can directly enforce the transformation of urothelial character to mesenchymal character and then promote the cell migration in bladder cancer cells.

## Discussion

In the most of cancer types, EP300 is believed to function as oncogene. For example, EP300 is required for the MycN expression in Neuroblastoma and it’s inhibitor shown a therapeutic potential [32]. Furthermore, another novel small molecule specifically targeting EP300 had been tested in hematologic malignancies [16]. In bladder cancer genomic analysis revealed that EP300 locus contained high mutation frequency and those mutation corelated to better prognosis [13,15], suggesting EP300 may function as oncogene in bladder cancer as well. In particularly, SNP of *EP300* (*R1627W*) was also believed as a tumor driver in bladder cancer [14]. Here, we reported the EP300 plays oncogenic role in bladder cancer, likely through driving migration of bladder cancer cells and upregulating of SNAI2-associated events.

SNAI2 is the pioneer transcriptional factor for the EMT event, it’s associated genes play critical role both in stemness and tumorigenesis [18]. Overexpression of SNAI2 forcedly driven epithelial cells into high migration capacity mesenchymal-like cells [33]. In pancreatic cancer, ESR1 was recruited upon the SNAI2 promoter, suggesting estrogen signaling promotes EMT event, although this study proposed the EP300/CBP complex may be involved, yet lack of directly evidence [17]. Our study confirmed that EP300 primarily exerts an oncogenic role in bladder cancer, particularly by regulating SNAI2 to drive cancer cell migration. In our data, known SNAI2-associated genes such as ELF3, FGF1, FERMT1, FGFBP1, IGF2, IGFBP3, SPARC and TP63 [34–38] were downregulated by A485 treatment (Supplementary table 1).

Regards to concentration, our study’s concentration of A485 aligned with the effective concentration range reported in multiple previous studies investigating A485 function in *in vitro* models [39]. This concentration has been shown to exhibit reliable biological activity in *in vitro* experiments. In our study, A485 (10μM) treatment for 24 hours significantly reduced H3K27ac levels in bladder cancer cells, while cell viability did not sharply decrease at this concentration (data not shown). This ensured that the observed migration phenotype resulted from specific EP300 inhibition rather than non-specific cytotoxicity. In *in vivo* studies of the hematopoietic system and prostate cancer have shown that a 100 mg/kg dose of A485 effectively inhibited tumor growth without significant observed toxicity. This dose is approximately 18 times higher than the *in vitro* experimental concentration [40,41]. Therefore, A485 is a relatively safe therapeutic candidate.

Although some studies suggested that EP300 may act as a tumor suppressor in certain cancers such as colorectal cancer [12], genomic analyses of bladder cancer often associate EP300 mutations with aberrant histone acetylation patterns. Our findings reveal that inhibiting EP300 significantly downregulated SNAI2 expression in bladder cancer cells and suppressed migration, supporting the potential of targeting EP300 for therapeutic interventions. Due to EP300 is an important epigenetic regulator, it also has broad functions associated with wide range of transcriptional factors, targeting EP300 inhibition may compromise by complex cascades rather than only by migration. In lung cancer, upregulation of EP300 inhibited STAT1 phosphorylation via SOCS1, leading to downregulation of antigen-presenting genes and helping cancer cells evaded immune surveillance. Therefore, the safety evaluation of EP300 inhibition under broader tumor immune microenvironment context still need to be further investigated in future.

To mitigate potential risks, future study need address following questions: ① To develop a bladder-specific delivery approach to enhance drug efficacy in tumor tissues; ② To explore combinatory or addictive therapeutic regimens to reduce chemotherapy resistance rate; ③ To identify biomarkers to precisely benefit to EP300-oncogenic patients.

Collectively, our work proved that oncogenic EP300 is required for SNAI2 expression and EP300-SNAI2 axis plays a critical role in the bladder cancer migration.

## Supporting information

S1 Fig Cell viability assay for T24 cells at 2h and 24h treatment. (TIF)

S1 Table A485 down-regulated gens in SW780 cells. SNAI2-related genes were highlighted in red. (TIF)

S2 Fig The original images of western blot results in the manuscript. A. Protein levels of EMT pathway-related proteins (E-cad, N-cad, Claudin-1, SNAI2, TWIST1, ZEB1), GAPDH, H3K27ac, and Histone H3 in T24 and SW780 cells treated with DMSO and A485. B. Protein levels of SNAI2 and GAPDH in four bladder cancer cell lines treated with DMSO and A485. C. Protein levels of SNAI2 and GAPDH in T24 cells transfected with vehicle and SNAI2 overexpressing vector. D, DMSO, A, A485, V, Vehicle, OE, OE-SANI2.
(PDF)
