# Supplementary figures and images for: EP300 promotes bladder cancer cell migration through SNAI2
Source: PLoS One. 2026 Jun 8;21(6):e0347209. doi: 10.1371/journal.pone.0347209 (PMC13245783; doi:10.1371/journal.pone.0347209)

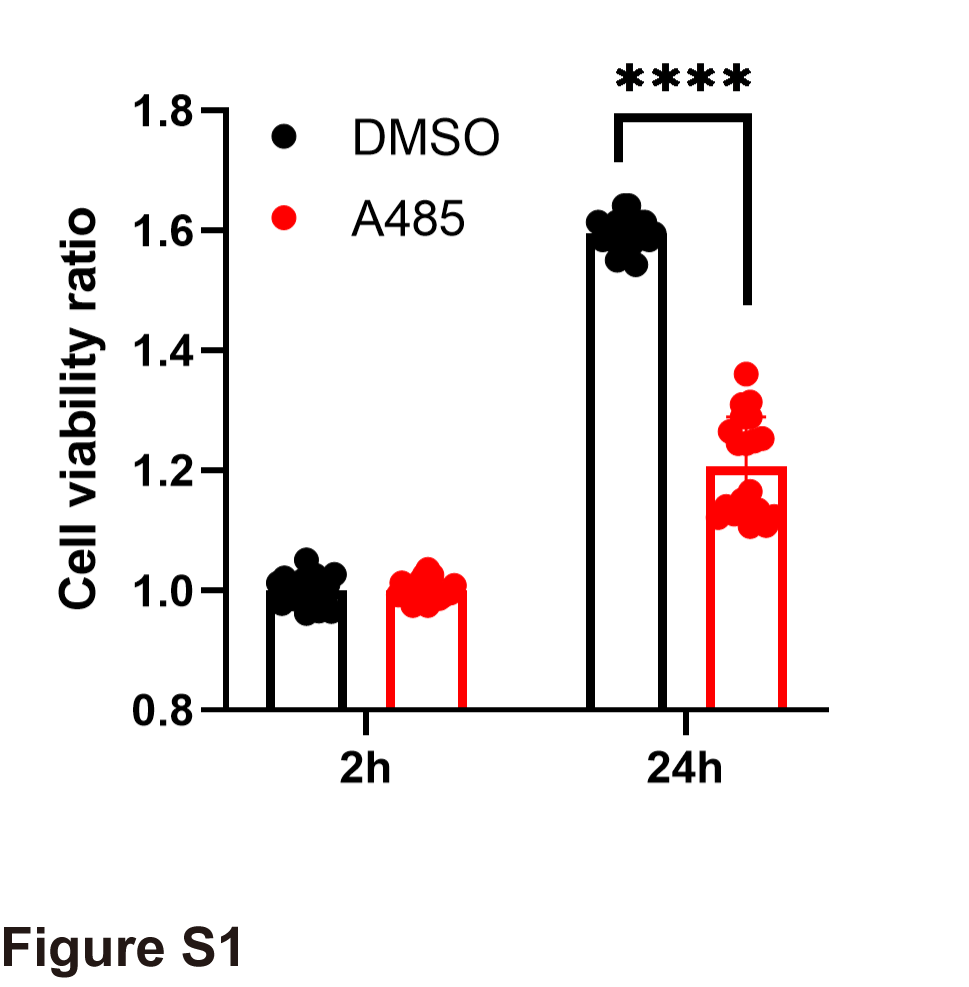

Supplement: S1 Fig — (TIF) [file pone.0347209.s001.tif]

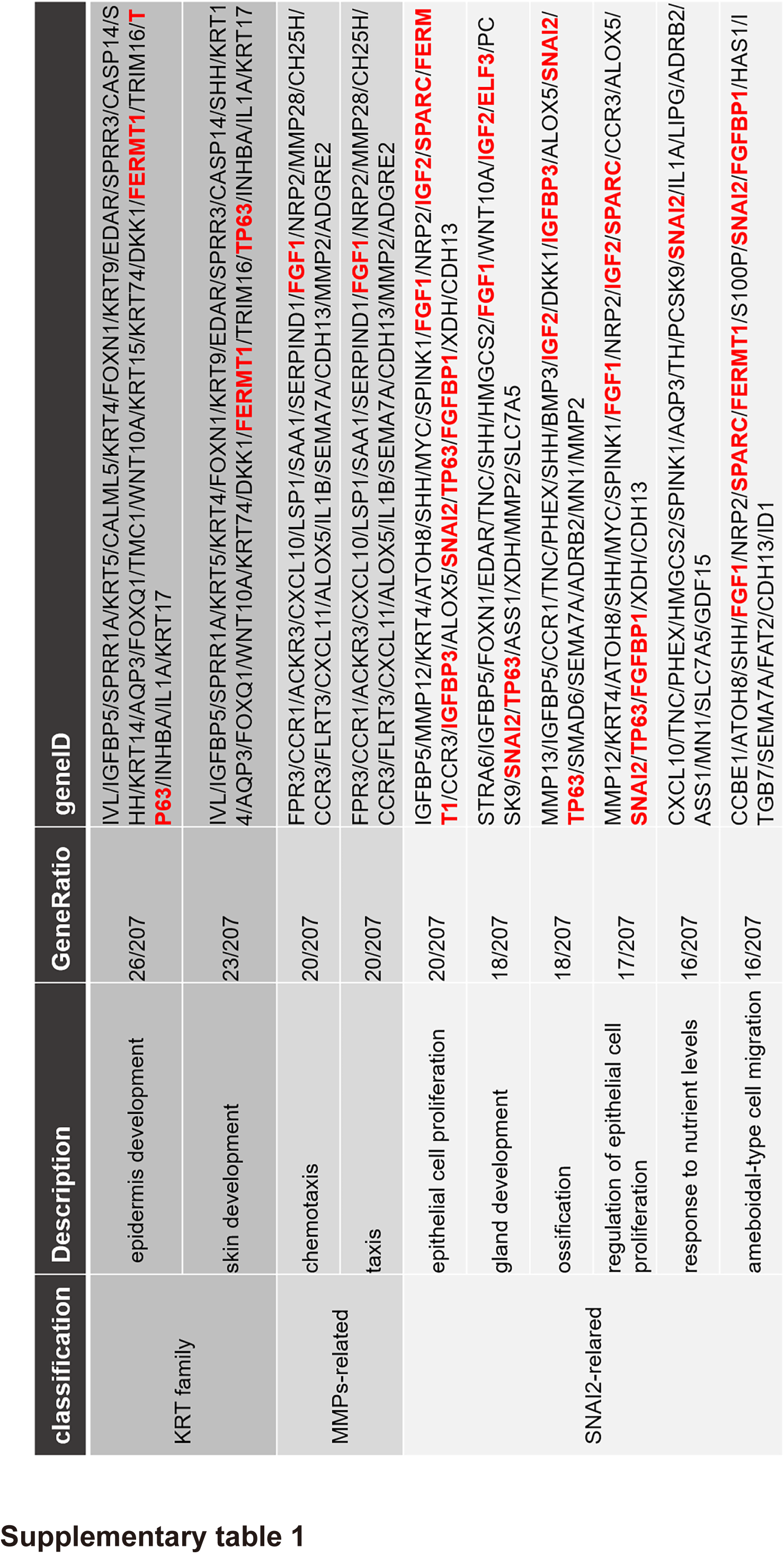

Supplement: S1 Table — (TIF) [file pone.0347209.s002.tif]
