# Supplementary material for: EP300 promotes bladder cancer cell migration through SNAI2
Source: PLoS One. 2026 Jun 8;21(6):e0347209. doi: 10.1371/journal.pone.0347209 (PMC13245783; doi:10.1371/journal.pone.0347209)
Supplement: S2 Fig — A. Protein levels of EMT pathway-related proteins (E-cad, N-cad, Claudin-1, SNAI2, TWIST1, ZEB1), GAPDH, H3K27ac, and Histone H3 in T24 and SW780 cells treated with DMSO and A485. B. Protein levels of SNAI2 and GAPDH in four bladder cancer cell lines treated with DMSO and A485. C. Protein levels of SNAI2 and GAPDH in T24 cells transfected with vehicle and SNAI2 overexpressing vector. D, DMSO, A, A485, V, Vehicle, OE, OE-SANI2. (PDF) [file pone.0347209.s003.pdf]

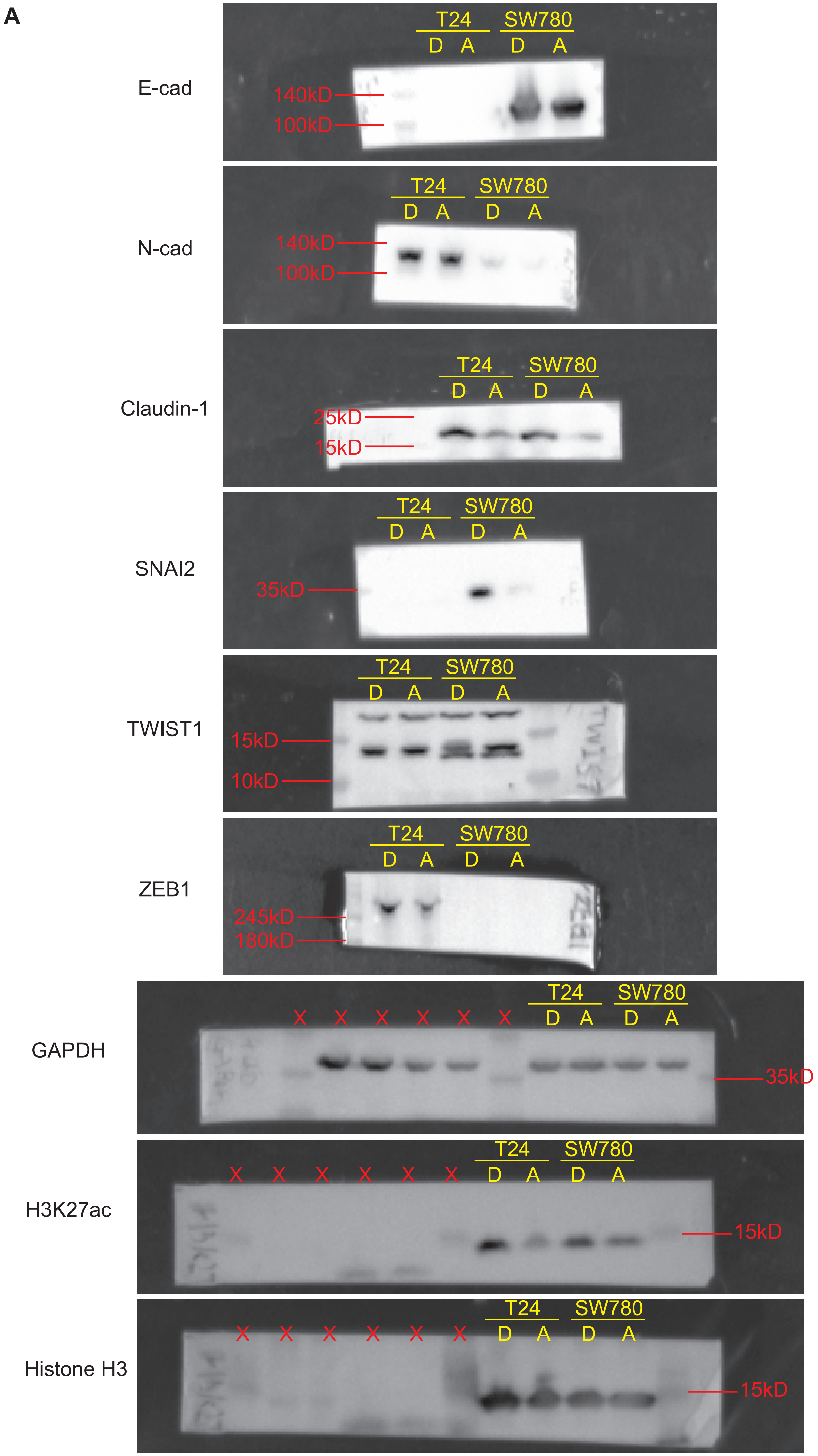

A
E-cad
T24
SW780
D A D A
140kD
100kD
N-cad
Claudin-1
25kD
15kD
SNAI2
35kD
TWIST1
15kD
10kD
ZEB1
245kD
180kD
GAPDH
35kD
H3K27ac
15kD
Histone H3
15kD

B

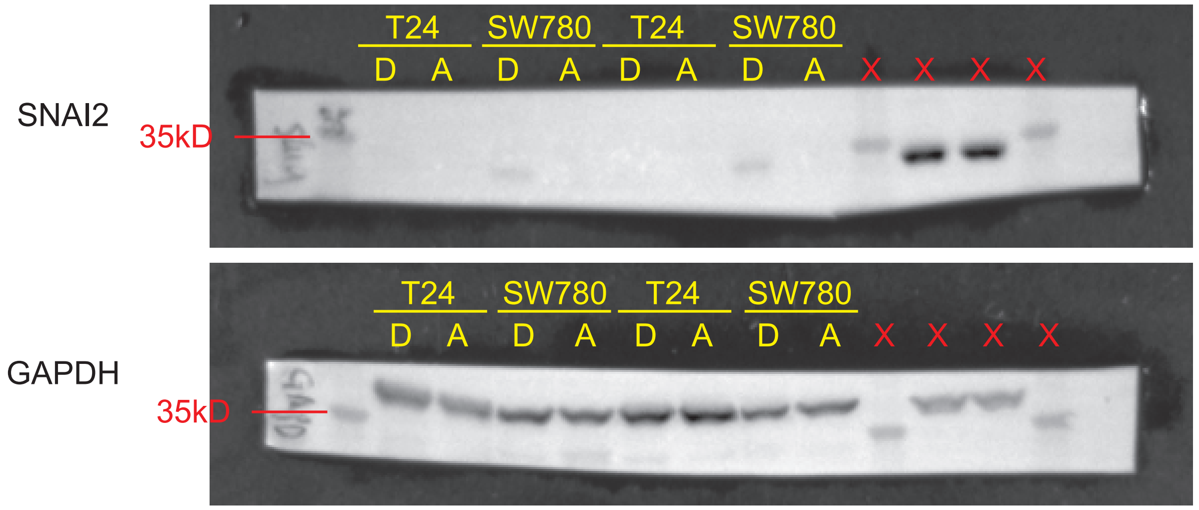

C

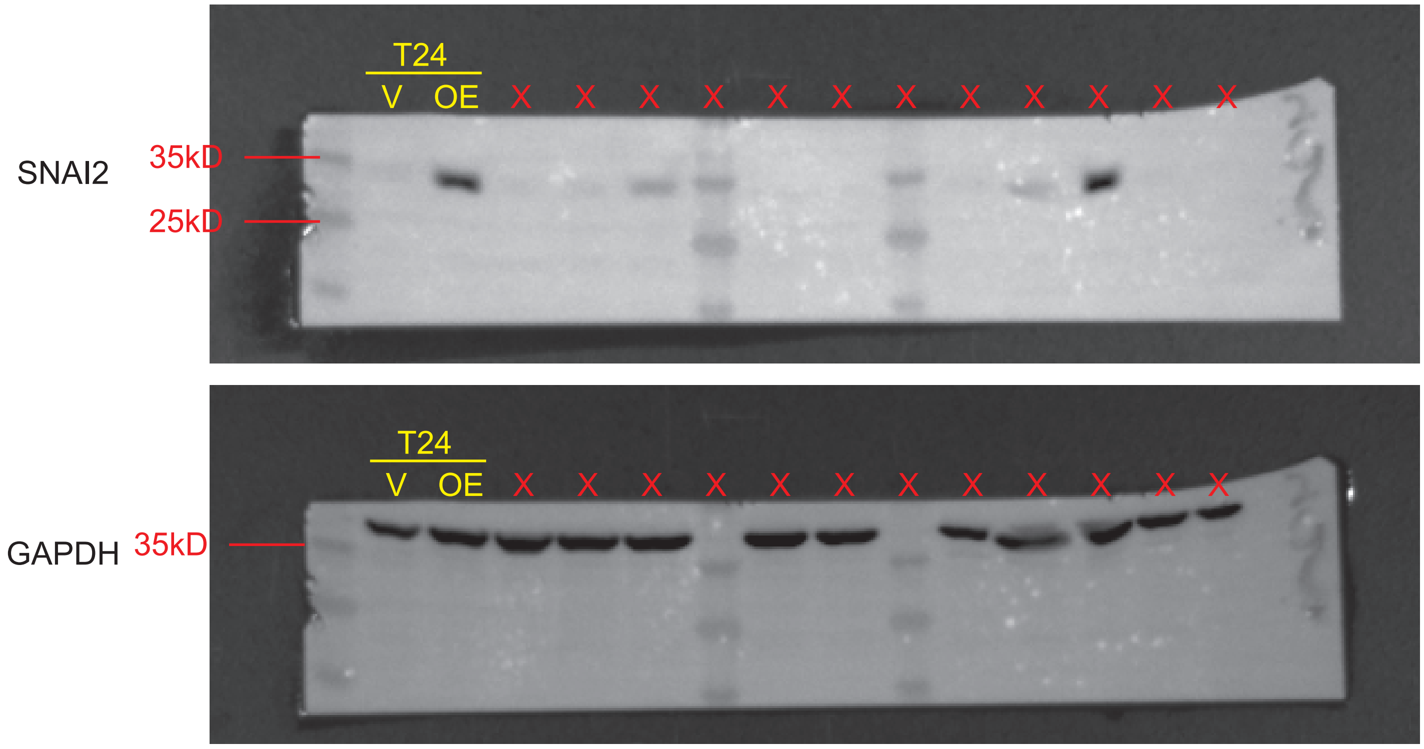

S1 images. The original images of western blot results in the manuscript. A. Protein levels of EMT pathway-related proteins (E-cad, N-cad, Claudin-1, SNAI2, TWIST1, ZEB1), GAPDH, H3K27ac, and Histone H3 in T24 and SW780 cells treated with DMSO and A485. B. Protein levels of SNAI2 and GAPDH in four bladder cancer cell lines treated with DMSO and A485. C. Protein levels of SNAI2 and GAPDH in T24 cells transfected with vehicle and SNAI2-overexpressing vector. D, DMSO, A, A485, V, Vehicle, OE,OE-SANI2.
