# Supplementary material for: EP300 promotes bladder cancer cell migration through SNAI2
Source: PLoS One. 2026 Jun 8;21(6):e0347209. doi: 10.1371/journal.pone.0347209 (PMC13245783; doi:10.1371/journal.pone.0347209)
Supplement: Attachment — Submitted filename: Response letter_Plos One .docx [file pone.0347209.s004.docx]

Response Letter to ` PLOS ONE ` Submission

Paper ID: PONE-D-25-21042

Paper Title: EP300 promotes bladder cancer cell migration through SNAI2

We want to thank you for your valuable comments.

We submitted 3 files, including a revised manuscript, a track changes file (to highlight differences between the revised and the original manuscript), and this file, i.e., the response letter (a complete response to the editor). In the following, we respond to each of your concerns and recommendations.

(EC: Editor’s Comment, RC: Reviewer’s Comment, AR: Author's Response)

Response to the Editor

**EC1:** Please ensure that your manuscript meets PLOS ONE's style requirements, including those for file naming. The PLOS ONE style templates can be found at

https://journals.plos.org/plosone/s/file?id=wjVg/PLOSOne_formatting_sample_main_body.pdf and

<https://journals.plos.org/plosone/s/file?id=ba62/PLOSOne_formatting_sample_title_authors_affiliations.pdf>

***Reply:*** *Thank you for your reminder.* *We have adjusted the style and file names of the manuscript according to the above requirements.*

**EC2:** PLOS ONE now requires that authors provide the original uncropped and unadjusted images underlying all blot or gel results reported in a submission’s figures or Supporting Information files. This policy and the journal’s other requirements for blot/gel reporting and figure preparation are described in detail at https://journals.plos.org/plosone/s/figures#loc-blot-and-gel-reporting-requirements and https://journals.plos.org/plosone/s/figures#loc-preparing-figures-from-image-files. When you submit your revised manuscript, please ensure that your figures adhere fully to these guidelines and provide the original underlying images for all blot or gel data reported in your submission. See the following link for instructions on providing the original image data: https://journals.plos.org/plosone/s/figures#loc-original-images-for-blots-and-gels.

In your cover letter, please note whether your blot/gel image data are in Supporting Information or posted at a public data repository, provide the repository URL if relevant, and provide specific details as to which raw blot/gel images, if any, are not available. Email us at plosone@plos.org if you have any questions.

***Reply:*** *Thank you for your asking. Our blot/gel image data are neither in Supporting Information nor posted at a public data repository.*

**EC3:** Thank you for stating the following in the Acknowledgments Section of your manuscript:
This work was supported by the National Natural Science Foundation of China No.32070818 to C.M.G., the Xingdian talent support program of Yunnan Province to C.M.G. and Guangdong Hybribio Biotech Funding No.H20230314, H20230311 and H20230313 to C.M.G.

We note that you have provided funding information that is not currently declared in your Funding Statement. However, funding information should not appear in the Acknowledgments section or other areas of your manuscript. We will only publish funding information present in the Funding Statement section of the online submission form.
Please remove any funding-related text from the manuscript and let us know how you would like to update your Funding Statement. Currently, your Funding Statement reads as follows:
Zhang Q. He C. and Guo C. are supported from National Natural Science Foundation of China No.32070818, Guangdong Hybribio Biotech Funding H20230314, H20230311 and H20230313.

Funders are not play any role in the study design, data collection and analysis, decision to publish, or preparation of the manuscript.

Please include your amended statements within your cover letter; we will change the online submission form on your behalf.

***Reply:*** *Thank for your suggestion. We removed funding information and modified the cover letter.*

**EC4:** Please include your full ethics statement in the ‘Methods’ section of your manuscript file. In your statement, please include the full name of the IRB or ethics committee who approved or waived your study, as well as whether or not you obtained informed written or verbal consent. If consent was waived for your study, please include this information in your statement as well.

***Reply:*** *Thank for your comments.* *We added the ethics statement in the methods section, please see Page 4, line 85-94. We included both human and animal information.*

**EC5:** Thank you for uploading your study's underlying data set. Unfortunately, the repository you have noted in your Data Availability statement does not qualify as an acceptable data repository according to PLOS's standards.

At this time, please upload the minimal data set necessary to replicate your study's findings to a stable, public repository (such as figshare or Dryad) and provide us with the relevant URLs, DOIs, or accession numbers that may be used to access these data. For a list of recommended repositories and additional information on PLOS standards for data deposition, please see <https://journals.plos.org/plosone/s/recommended-repositories>.

***Reply:*** *Thank for your comments. We added the uploading data information in the current revision. Please see page 10, line 203-205; 216-217.*

**EC6:** Please amend the manuscript submission data (via Edit Submission) to include author Tao Huang

***Reply:*** *Thank for your comments. We corrected this in the current submission.*

**EC7:** If the reviewer comments include a recommendation to cite specific previously published works, please review and evaluate these publications to determine whether they are relevant and should be cited. There is no requirement to cite these works unless the editor has indicated otherwise.

***Reply:*** *Thank for your kindly reminder. Our decisions all bases on Truth, at least what we believe.*

**Reviewer #1**

**RC1.0:** This manuscript investigates the mechanism by which EP300 promotes bladder cancer cell migration through SNAI2. The study design is reasonable, the experimental data are relatively sufficient, and the conclusions have certain scientific significance. After review, it is recommended to "Accept after minor revisions".

**AC1.0:** *We thank the reviewer for the kind comments and hopefully we could push the field forward a little bit.*

**RC1.1:** In the cell culture section, the source background information of the four bladder cancer cell lines (SW780, T24, RT4, and 5637) should be clarified, including their pathological grades, metastatic potentials, etc., to enhance the rationality of the selection of experimental models.

**AC1.1:** *Thank you for your suggestion, according to your suggestion, we added the information you suggested. The added information is as follows (see details in page 5, lines 105-110 of the revised manuscript):*

*Add:" Among them, RT4 (derived from bladder papillary transitional cell carcinoma) and SW780 (derived from bladder transitional cell carcinoma) exhibit low malignancy, 5637 (derived from Grade II bladder cancer) shows intermediate malignancy, while T24 (derived from metastatic bladder cell adenocarcinoma) demonstrates the highest malignancy level."*

**RC1.2:** In the scratch wound healing assay, it is necessary to supplement the specific composition of the "serum-free complete medium" and the reason for choosing t-his condition for the experiment; in the Transwell assay, the reason for using the same concentration of A485 in both the upper and lower chambers should be clarified.

**AC1.2:** *Thank you for your suggestion, according to your suggestion, we added the information you suggested. The added information is as follows* (see details in page 7, 8, *lines 156-171* of the revised manuscript)*:*

*Add:" To exclude the wound-healing effects caused by serum-induced cell proliferation, the cells were then cultured in serum-free complete medium supplemented with either DMSO or A485 (10μM). For T24 cells, the serum-free complete medium consisted of McCoy's 5A and 1% penicillin/streptomycin, while for SW780 cells, it was composed of 1640 medium, 1% penicillin/streptomycin, and 1% HEPES." (see details in lines 138-143 of the revised manuscript)*

*Add: "To investigate whether A485 can inhibit the migration ability of bladder cancer cells rather than the chemotactic effect of the drug on the cells, the same concentration of DMSO or A485 was added to both the upper and lower chambers. "*

**RC1.3:** Regarding the improvement of result analysis:
It is mentioned in Figure 1 that "ac-EP300 is significantly increased in 2/4 tumor tissues". Specific statistical analysis results, including sample size and P-value, should be supplemented to more strongly support the association between EP300 activation and the malignant progression of bladder cancer.
**AC1.3:** *Thank you for your suggestion, we have updated the samples of 4 patients to 12 patients and added statistical results with significant differences of EP300 protein level. (details of the tissue chips are shown page 4, 82-85, details of the results are shown in page10,11 in lines 222-227).*

*Add:" The bladder cancer tissues from the patients were obtained from the Second Affiliated Hospital of Kunming Medical University, and all studies were performed according to protocols reviewed and approved by Medical Ethics Committee: FEY-BG-39-2.0/审-PJ-科-2023-246, FEY-BG-38-1.2/审-YJ-2024-114.”*

*Add:" To investigate the correlation between EP300 and human bladder cancer. We generated human tissue microarray chips, including 12 pairs of bladder cancer and adjacent tissues. Then we performed immunohistochemical staining for EP300 on array chips (Figure 1A). We evaluated the expression level of EP300 in each tissue sample as ranging from 0 to 4 points based on the degree of staining of nuclear EP300, and conducted statistical analysis (Fig 1B).”*

**RC1.4:** In the RNAseq analysis, for the 229 commonly downregulated genes, their specific functional classifications and direct evidence of association with SNAI2-related pathways should be supplemented, such as whether there are target genes of SNAI2.

**AC1.4:** *Thank you for your suggestion, we have added a table in the supplementary data, SNAI2 and it’s known downstream genes are discussed in page 16, 347-351.*

*Add:" In our data, known SNAI2-associated genes such as ELF3, FGF1, FERMT1, FGFBP1, IGF2, IGFBP3, SPARC and TP63 [34-38] were downregulated by A485 treatment.”*

**RC1.5:** Regarding the deepening of the discussion section:
The discussion mentions that EP300 may have oncogenic or tumor-suppressive effects in different cancers. Combined with the results of this study, it is necessary to further elaborate on the specificity and potential risks of targeting EP300 as a therapeutic strategy in bladder cancer.

**AC1.5:** *Thank you for your valuable comments and insightful perspectives on this paper. The two aspects you pointed out are crucial for deepening the substance of this study and its clinical translational potential. We have revised and supplemented the manuscript accordingly based on your suggestions, with specific explanations provided below: (see details in page 17,18 lines 364-382 of the revised manuscript)*

*Add: “Although some studies suggested that EP300 may act as a tumor suppressor in certain cancers such as colorectal cancer [12], genomic analyses of bladder cancer often associate EP300 mutations with aberrant histone acetylation patterns. Our findings reveal that inhibiting EP300 significantly downregulated SNAI2 expression in bladder cancer cells and suppressed migration, supporting the potential of targeting EP300 for therapeutic interventions. Due to EP300 is an important epigenetic regulator, it also has broad functions associated with wide range of transcriptional factors, targeting EP300 inhibition may compromise by complex cascades rather than only by migration. In lung cancer, upregulation of EP300 inhibited STAT1 phosphorylation via SOCS1, leading to downregulation of antigen-presenting genes and helping cancer cells evaded immune surveillance. Therefore, the safety evaluation of EP300 inhibition under broader tumor immune microenvironment context still need to be further investigated in future.*

*To mitigate potential risks, future study need address following questions: ① To develop a bladder-specific delivery approach to enhance drug efficacy in tumor tissues; ② To explore combinatory or addictive therapeutic regimens to reduce chemotherapy resistance rate; ③ To identify biomarkers to precisely benefit to EP300-oncogenic patients. ”*

**RC1.6:** The basis for selecting the concentration of A485 (10μM) in this study should be supplemented, such as whether it has been verified by pre-experiments, as well as references regarding the possible safety and effectiveness of this concentration in vivo.

**AC1.6:** *Thank you for your valuable comments and insightful perspectives on this paper. We have revised and supplemented the manuscript accordingly in discussion, with specific explanations provided below: (see details in page 16, 17 in lines 351-363 of the revised manuscript)*

Add:” *Regards to concentration, our study’s concentration of A485 aligned with the effective concentration range reported in multiple previous studies investigating A485 function in in vitro models [39]. This concentration has been shown to exhibit reliable biological activity in in vitro experiments. In our study, A485 (10μM) treatment for 24 hours significantly reduced H3K27ac levels in bladder cancer cells, while cell viability did not sharply decrease at this concentration (data not shown). This ensured that the observed migration phenotype resulted from specific EP300 inhibition rather than non-specific cytotoxicity. In in vivo studies of the hematopoietic system and prostate cancer have shown that a 100 mg/kg dose of A485 effectively inhibited tumor growth without significant observed toxicity. This dose is approximately 18 times higher than the in vitro experimental concentration [40, 41]. Therefore, A485 is a relatively safe therapeutic candidate.*”

**RC1.7:** Regarding data availability and ethical statements:
The data availability section mentions that all data are fully available without restriction. Specific access methods, such as database names and accession numbers, should be supplemented.
In the ethical approval section, it is necessary to clarify whether informed consent was obtained from the participants during the collection of human samples and the form of consent (written or oral).

**AC1.7:** *Thank you for your comments. We added the uploading data information in the current revision. Please see page 10, line 203-205; 216-217.*

Reviewer #2

**RC2.0:** This study investigates the role of EP300 in bladder cancer migration. It confirms the positive association between EP300 and bladder malignancy. The study observed A485, which is a small-molecule inhibitor of EP300, significantly inhibited the migration of multiple bladder cancer cell lines (SW780, T24, RT4, 5637) both in scratch wound healing and Transwell assays, and disrupted organoid migration in 3D culture. Using bulk RNA-seq analysis and western blotting the research found that A485 downregulates SNAI2-related signaling pathways and reduce the expression of SNAI2 and the H3K27ac level. Overexpression of SNAI2 enhances the bladder cancer cell migration, and EP300 can promote bladder cancer cell migration via SNAI2, and targeting EP300 could be a potential therapeutic strategy.

**AC2.0:** *Thank you for your careful evaluation of this manuscript.*

**RC2.1:** Lack of in vivo validation for the effects of EP300 inhibition. Effects the translational medical value of the conclusion.

**AC2.1:** *Thank you for your valuable comments. We deeply agree with you, therefore we added the in vivo validation for EP300 inhibition by BBN-induced spontaneous bladder cancer model in revised version, please see page Page 12-13, line 263-279*

Add:”

*To investigate the effects of A485 in vivo, we established a BBN-induced mouse bladder cancer model. Mice were continuously treated with BBN for five months, while DMSO or A485 treatment began two months after BBN administration and continued until the end of the experiment. Pathological evaluation of the extent of bladder cancer invasion was performed after the experiment (Fig. 4A). Hematoxylin and eosin (HE) staining results showed that bladder cancer in the DMSO group invaded the muscle layer, whereas in the A485 group, invasion was limited to the lamina propria, indicating a reduced degree of invasion after A485 treatment (Fig. 4B). We further quantified the proportion of different invasion types in each bladder epithelium. The results revealed a decreasing trend in the proportion of invasive types (lamina propria-invasive and muscle-invasive) after A485 treatment, although the difference was not statistically significant (Fig. 4C). Immunohistochemical staining results also demonstrated a decrease in H3K27ac levels in bladder epithelial cells (marked by KRT5) after A485 treatment (Fig. 4D), suggesting that the effect of A485 on the extent of bladder cancer invasion in mice is associated with H3K27ac.”*

**RC2.2:** The results only used 4 matched pairs of bladder cancer and adjacent tissues. Is the sample size too small?

**AC2.1:** *Thank you for your suggestion, we have updated the samples of 4 patients to 12 patients and added statistical results with significant differences of EP300 protein level. (details of the tissue chips are shown page 4, 82-85, details of the results are shown in page10,11 in lines 222-227).*

*Add:" The bladder cancer tissues from the patients were obtained from the Second Affiliated Hospital of Kunming Medical University, and all studies were performed according to protocols reviewed and approved by Medical Ethics Committee: FEY-BG-39-2.0/审-PJ-科-2023-246, FEY-BG-38-1.2/审-YJ-2024-114.”*

*Add:" To investigate the correlation between EP300 and human bladder cancer. We generated human tissue microarray chips, including 12 pairs of bladder cancer and adjacent tissues. Then we performed immunohistochemical staining for EP300 on array chips (Figure 1A). We evaluated the expression level of EP300 in each tissue sample as ranging from 0 to 4 points based on the degree of staining of nuclear EP300, and conducted statistical analysis (Fig 1B).”*

**RC2.3:** The discussion says that the EP300 has two opposite effects, but the study only explores the EP300’s role of promoting migratory, and don’t delve into the possibility of EP300 suppressing the bladder cancer, nor does it analyze the influence of these opposite effects for EP300-targeted therapeutic strategies.

**AC2.3:** *Thank you for your valuable comments. To evaluate the translational potential, we performed the current newly finished in vivo evaluation, in our data, we observed that EP300 inhibition indeed attenuated bladder cancer progression, however due to BBN-model contained wild variance, we had not collected the significance at this moment. Please see* **AC2.1.**

*In addition, we have revised and supplemented the manuscript to discuss the dual role of EP300 in different cancer types (see details in page 17,18 lines 364-382 of the revised manuscript)*

*Add: “Although some studies suggested that EP300 may act as a tumor suppressor in certain cancers such as colorectal cancer [12], genomic analyses of bladder cancer often associate EP300 mutations with aberrant histone acetylation patterns. Our findings reveal that inhibiting EP300 significantly downregulated SNAI2 expression in bladder cancer cells and suppressed migration, supporting the potential of targeting EP300 for therapeutic interventions. Due to EP300 is an important epigenetic regulator, it also has broad functions associated with wide range of transcriptional factors, targeting EP300 inhibition may compromise by complex cascades rather than only by migration. In lung cancer, upregulation of EP300 inhibited STAT1 phosphorylation via SOCS1, leading to downregulation of antigen-presenting genes and helping cancer cells evaded immune surveillance. Therefore, the safety evaluation of EP300 inhibition under broader tumor immune microenvironment context still need to be further investigated in future.*

**RC2.4:** The discussion only repeats the results of the study, lacks an analysis of the limitation of the study.
**AC2.4:** *Thank you for your valuable comments. We rewrote the discussion part and included the limitation of the study. (see details in page 18, lines 378-382 of the revised manuscript)*

*Add: “To mitigate potential risks, future study need address following questions: ① To develop a bladder-specific delivery approach to enhance drug efficacy in tumor tissues; ② To explore combinatory or addictive therapeutic regimens to reduce chemotherapy resistance rate; ③ To identify biomarkers to precisely benefit to EP300-oncogenic patients. ”*

RC2.5: Figure5, only inhibition of EP300 via A485 alone led to a decrease in SNAI2 expression was observed. Lack the direct evidence to demonstrate that EP300 binds to the promoter or enhancer region of SNAI2. It is recommended to supplement the study with experiments (such as ChIP-qPCR and luciferase reporter assay) to validate the regulatory relationship.

**AC2.5:** *Thank you for your valuable comments. We performed CUT&Tag ChIPseq, we found H3K27ac level was decreased after A485 treatment. (see details in page 9-10, lines 194-217 and in page 15 lines 313-320 of the revised manuscript)*

*Add: “H3K27ac, the most well-known consequence of histone acetyltransferase activity of EP300, was reported to be associated with enhancer activation[31]. To verify the direct regulatory of EP300 on SNAI2, we performed CUT&Tag assays of H3K27ac in SW780 cells. The results showed significant enrichment of H3K27ac at the SNAI2 gene body and upstream region approximately 4 Kb away, which decreased after A485 treatment, suggesting that EP300 may regulate SNAI2 transcription through enhancer-activity (Figure 6C). “*

**RC2.6:** PLOS authors have the option to publish the peer review history of their article ([what does this mean?](https://journals.plos.org/plosone/s/editorial-and-peer-review-process#loc-peer-review-history)). If published, this will include your full peer review and any attached files.
If you choose “no”, your identity will remain anonymous but your review may still be made public.
**AC2.6:** *No, thanks.*
