# Supplementary material for: EP300 promotes bladder cancer cell migration through SNAI2
Source: PLoS One. 2026 Jun 8;21(6):e0347209. doi: 10.1371/journal.pone.0347209 (PMC13245783; doi:10.1371/journal.pone.0347209)
Supplement: Attachment — Submitted filename: +Response letter_Plos One-R2.docx [file pone.0347209.s005.docx]

**Reply to Reviewers' Comments**

We are deeply grateful to the reviewers for their insightful and constructive feedback on our manuscript entitled "EP300 promotes bladder cancer cell migration through SNAI2." The critiques have been invaluable in helping us strengthen the evidence and clarify the presentation of our study. Below, we provide a point-by-point response to each comment. All planned revisions and additions to the manuscript are detailed herein, and corresponding changes will be clearly highlighted in the revised manuscript.

### ****Response to Reviewer #1****

- How can you demonstrate that EP300 directly regulates SNAI2, rather than through indirect pathways? The use of inhibitors and H3K27ac CUT&Tag alone is insufficient to prove direct regulation. Can you provide EP300-specific ChIP experimental data? Furthermore, can SNAI2 overexpression fully reverse the inhibitory effect of A485 on migration, thereby proving it is the key downstream effector of EP300?

**Reply:** We thank the reviewer for this crucial suggestion to deepen our mechanistic investigation. We agree that complementing the CUT&Tag data with direct binding evidence and a functional rescue experiment is essential.

1. **EP300 CUT&Tag:** We performed CUT&Tag assays using a EP300-specific antibody in SW780 cells. This experiment provided direct evidence of EP300 recruitment to the SNAI2 locus. These results were added to results section (Page 16, Lines 351-354) and a new Figure 7 (Page 29, Line 589).
2. **SNAI2 Overexpression Rescue Experiment:** We conducted a key rescue experiment by transfecting an expression vector for SNAI2 (or using a lentivirus) into bladder cancer cells, followed by treatment with A485. We then assessed whether forced expression of SNAI2 can counteract the inhibitory effects of A485 on cell migration in wound healing/Transwell assays. This experiment is critical to establish SNAI2 as a major functional downstream effector of EP300. The results were added to results section (Page 17, Lines 356-359) and a new Figure 7 (Page 29, Line 589).

- The entire study heavily depends on a single inhibitor, A485. To exclude potential off-target effects, can you provide genetic evidence (e.g., EP300 knockdown or knockout) demonstrating that the observed phenotypes (inhibited migration and downregulated SNAI2) are consistent with pharmacological inhibition?

**Reply:** To consolidate our conclusions and rule out potential off-target effects of A485, we attempted to knockout EP300 in T24 and SW780 cells using sgRNA, but these efforts were unsuccessful. Therefore, we supplemented the study by examining the effects of small-molecule inhibitors targeting different domains of EP300—namely A485, C646, and CBP30—on the migratory ability of T24 cells, thereby enhancing the specificity assessment of A485. These data were showed in a new Figure 3 (Page 25, Line 559) and results section (Page 13, Lines 267-279).

- The manuscript states that A485 only shows a 'trend' *in vivo* without statistical significance. Under these circumstances, why is this still presented as key supporting evidence? Please provide detailed sample sizes (n values) and statistical methods, and discuss the impact of this non-significant result on your conclusions.

**Reply:** We apologize for the lack of clarity and appropriate framing. The reviewer is correctly highlighted this weakness.

We clearly stated the sample size (n=4 per group) in figure legend for the *in vivo* BBN model.

In the previous manuscript, we performed a combined analysis of the proportions of different infiltration types with or without A485 treatment using the Two-way ANOVA statistical method; however, no significant differences were observed. Therefore, we updated the data presentation and statistical approach, employing the Unpaired t-test to analyze individual infiltration types separately. The results demonstrated that after A485 treatment, the proportion of normal bladder epithelium in mice significantly increased, while the proportion of tumors infiltrating the lamina propria significantly decreased, providing robust evidence for our conclusions. The results are presented in a new Figure 5 (Page 27, Line 573) and results section (Pages 14-15, lines 308-311).

- How have you verified that the inhibition of migration by A485 is not simply due to cytotoxicity or proliferation suppression? Please provide cell viability/proliferation data under the exact same conditions used in the migration assays as supplementary information.

**Reply:** According to ATCC information, the mitotic time of T24 cells is approximately 12 hours. To exclude the potential confounding effect of A485 on bladder cancer cell proliferation that could interfere with the interpretation of migration results, we supplemented the original 24‑hour migration data with 6‑hour and 12‑hour Transwell assays. The results show that after A485 treatment, the migration ability of T24 cells became significantly reduced starting at 6 hours and declined by 1.796-fold at 24h treatment. In cell viability assay, A485 had no effect on viability at 2h treatment yet it reduced cell proliferation by 0.389-fold at 24h treatment under the same conditions for Transwell (Figure S1), indicating that the inhibitory effect of A485 in the Transwell assay is majorly independent of cell proliferation. These results are presented in the new Figure 2 (Page 24, Line 550), Figure S1 (Page 29, Line 591 ), results section (Page 11-12, Lines 243-252) and Methods (Page 9, Lines 182-191 )

- Please comprehensively check and correct all figure citations throughout the manuscript.

**Reply:** Thank you for this meticulous check. We have modified the citation numbers to superscript format throughout the entire manuscript.

### ****Response to Reviewer #2****

- First, per PLOS ONE’s mandates, uncropped, unadjusted blot/gel images (e.g., Western blot for SNAI2 and H3K27ac) remain unpublished—please deposit these in the supplementary materials or a public repository.

**Reply:** We thank the reviewer for reminding us of this important journal policy. We have uploaded a **PDF file named "** **S1_raw_images"** including the full, uncropped scans of all Western blots and gels presented in the manuscript (SNAI2, H3K27ac, EP300, and loading controls).

- Second, the in vivo BBN model shows a trend of reduced invasion but lacks statistical significance, possibly due to small sample size (n=4 per group); expanding the cohort or adding supplementary functional assays (e.g., metastasis tracking) would reinforce translational relevance.

**Reply:** We appreciate the reviewer's constructive suggestion to strengthen the in vivo validation.

 As also addressed in response to Reviewer #1 (Point 3), we clearly stated the sample size limitation and the resulting lack of statistical power in the current BBN experiment. **In the previous manuscript, we performed a combined analysis of the proportions of different infiltration types with or without A485 treatment using the Two-way ANOVA statistical method; however, no significant differences were observed. Therefore, we updated the data presentation and statistical approach, employing the Unpaired t-test to analyze individual infiltration types separately. The results demonstrated that after A485 treatment, the proportion of normal bladder epithelium in mice significantly increased, while the proportion of tumors infiltrating the lamina propria significantly decreased, providing robust evidence for our conclusions. The results are presented** in a new Figure 5 (Page 27, Line 573) and results section (Pages 14-15, lines 308-311).

We are committed to making these revisions and thank the reviewers once again for their expert guidance.
