# Supplementary material for: EP300 promotes bladder cancer cell migration through SNAI2
Source: PLoS One. 2026 Jun 8;21(6):e0347209. doi: 10.1371/journal.pone.0347209 (PMC13245783; doi:10.1371/journal.pone.0347209)
Supplement: Peer review history

# Peer review: Decision Letter 0

**DOI:** 10.1371/journal.pone.0347209.r001

**Authors:** Chien-Feng Li

7 Sep 2025

PONE-D-25-21042

EP300 promotes bladder cancer cell migration through SNAI2

PLOS ONE

Dear Dr. Guo,

Thank you for submitting your manuscript to PLOS ONE. After careful consideration, we feel that it has merit but does not fully meet PLOS ONE’s publication criteria as it currently stands. Therefore, we invite you to submit a revised version of the manuscript that addresses the points raised during the review process.

==============================

**ACADEMIC EDITOR:**

Dear Authors,

We invite you to revise your work based on the reviewers' comments.

==============================

Please submit your revised manuscript by Oct 22 2025 11:59PM. If you will need more time than this to complete your revisions, please reply to this message or contact the journal office at plosone@plos.org. When you're ready to submit your revision, log on to https://www.editorialmanager.com/pone/ and select the 'Submissions Needing Revision' folder to locate your manuscript file.

Please include the following items when submitting your revised manuscript:

- A rebuttal letter that responds to each point raised by the academic editor and reviewer(s). You should upload this letter as a separate file labeled 'Response to Reviewers'.
- A marked-up copy of your manuscript that highlights changes made to the original version. You should upload this as a separate file labeled 'Revised Manuscript with Track Changes'.
- An unmarked version of your revised paper without tracked changes. You should upload this as a separate file labeled 'Manuscript'.

If you would like to make changes to your financial disclosure, please include your updated statement in your cover letter. Guidelines for resubmitting your figure files are available below the reviewer comments at the end of this letter.

If applicable, we recommend that you deposit your laboratory protocols in protocols.io to enhance the reproducibility of your results. Protocols.io assigns your protocol its own identifier (DOI) so that it can be cited independently in the future. For instructions see: https://journals.plos.org/plosone/s/submission-guidelines#loc-laboratory-protocols. Additionally, PLOS ONE offers an option for publishing peer-reviewed Lab Protocol articles, which describe protocols hosted on protocols.io. Read more information on sharing protocols at https://plos.org/protocols?utm_medium=editorial-email&utm_source=authorletters&utm_campaign=protocols.

We look forward to receiving your revised manuscript.

Kind regards,

Chien-Feng Li, M.D., Ph.D.

Academic Editor

PLOS ONE

Journal Requirements:

When submitting your revision, we need you to address these additional requirements.

1. Please ensure that your manuscript meets PLOS ONE's style requirements, including those for file naming. The PLOS ONE style templates can be found at

https://journals.plos.org/plosone/s/file?id=wjVg/PLOSOne_formatting_sample_main_body.pdf and

https://journals.plos.org/plosone/s/file?id=ba62/PLOSOne_formatting_sample_title_authors_affiliations.pdf

2. PLOS ONE now requires that authors provide the original uncropped and unadjusted images underlying all blot or gel results reported in a submission’s figures or Supporting Information files. This policy and the journal’s other requirements for blot/gel reporting and figure preparation are described in detail at https://journals.plos.org/plosone/s/figures#loc-blot-and-gel-reporting-requirements and https://journals.plos.org/plosone/s/figures#loc-preparing-figures-from-image-files. When you submit your revised manuscript, please ensure that your figures adhere fully to these guidelines and provide the original underlying images for all blot or gel data reported in your submission. See the following link for instructions on providing the original image data: https://journals.plos.org/plosone/s/figures#loc-original-images-for-blots-and-gels.

In your cover letter, please note whether your blot/gel image data are in Supporting Information or posted at a public data repository, provide the repository URL if relevant, and provide specific details as to which raw blot/gel images, if any, are not available. Email us at plosone@plos.org if you have any questions.

3. Thank you for stating the following in the Acknowledgments Section of your manuscript:

This work was supported by the National Natural Science Foundation of China No.32070818 to C.M.G., the Xingdian talent support program of Yunnan Province to C.M.G. and Guangdong Hybribio Biotech Funding No.H20230314, H20230311 and H20230313 to C.M.G.

We note that you have provided funding information that is not currently declared in your Funding Statement. However, funding information should not appear in the Acknowledgments section or other areas of your manuscript. We will only publish funding information present in the Funding Statement section of the online submission form.

Please remove any funding-related text from the manuscript and let us know how you would like to update your Funding Statement. Currently, your Funding Statement reads as follows:

Zhang Q. He C. and Guo C. are supported from National Natural Science Foundation of China No.32070818, Guangdong Hybribio Biotech Funding H20230314, H20230311 and H20230313.

Funders are not play any role in the study design, data collection and analysis, decision to publish, or preparation of the manuscript.

Please include your amended statements within your cover letter; we will change the online submission form on your behalf.

4. Please include your full ethics statement in the ‘Methods’ section of your manuscript file. In your statement, please include the full name of the IRB or ethics committee who approved or waived your study, as well as whether or not you obtained informed written or verbal consent. If consent was waived for your study, please include this information in your statement as well.

5. Thank you for uploading your study's underlying data set. Unfortunately, the repository you have noted in your Data Availability statement does not qualify as an acceptable data repository according to PLOS's standards.

At this time, please upload the minimal data set necessary to replicate your study's findings to a stable, public repository (such as figshare or Dryad) and provide us with the relevant URLs, DOIs, or accession numbers that may be used to access these data. For a list of recommended repositories and additional information on PLOS standards for data deposition, please see https://journals.plos.org/plosone/s/recommended-repositories.

6. Please amend the manuscript submission data (via Edit Submission) to include author Tao Huang

7. If the reviewer comments include a recommendation to cite specific previously published works, please review and evaluate these publications to determine whether they are relevant and should be cited. There is no requirement to cite these works unless the editor has indicated otherwise.

[Note: HTML markup is below. Please do not edit.]

Reviewers' comments:

Reviewer's Responses to Questions

**Comments to the Author**

1. Is the manuscript technically sound, and do the data support the conclusions?

The manuscript must describe a technically sound piece of scientific research with data that supports the conclusions. Experiments must have been conducted rigorously, with appropriate controls, replication, and sample sizes. The conclusions must be drawn appropriately based on the data presented.

Reviewer #1: Yes

Reviewer #2: Yes

**********

2. Has the statistical analysis been performed appropriately and rigorously?

Reviewer #1: Yes

Reviewer #2: Yes

**********

3. Have the authors made all data underlying the findings in their manuscript fully available?

The PLOS Data policy requires authors to make all data underlying the findings described in their manuscript fully available without restriction, with rare exception (please refer to the Data Availability Statement in the manuscript PDF file). The data should be provided as part of the manuscript or its supporting information, or deposited to a public repository. For example, in addition to summary statistics, the data points behind means, medians and variance measures should be available. If there are restrictions on publicly sharing data—e.g. participant privacy or use of data from a third party—those must be specified.

Reviewer #1: Yes

Reviewer #2: Yes

**********

4. Is the manuscript presented in an intelligible fashion and written in standard English?

PLOS ONE does not copyedit accepted manuscripts, so the language in submitted articles must be clear, correct, and unambiguous. Any typographical or grammatical errors should be corrected at revision, so please note any specific errors here.

Reviewer #1: Yes

Reviewer #2: Yes

**********

5. Review Comments to the Author

Please use the space provided to explain your answers to the questions above. You may also include additional comments for the author, including concerns about dual publication, research ethics, or publication ethics. (Please upload your review as an attachment if it exceeds 20,000 characters)

Reviewer #1: This manuscript investigates the mechanism by which EP300 promotes bladder cancer cell migration through SNAI2. The study design is reasonable, the experimental data are relatively sufficient, and the conclusions have certain scientific significance. After review, it is recommended to "Accept after minor revisions". The specific revision suggestions are as follows:

Regarding the supplementation of experimental details:

In the cell culture section, the source background information of the four bladder cancer cell lines (SW780, T24, RT4, and 5637) should be clarified, including their pathological grades, metastatic potentials, etc., to enhance the rationality of the selection of experimental models.

In the scratch wound healing assay, it is necessary to supplement the specific composition of the "serum-free complete medium" and the reason for choosing this condition for the experiment; in the Transwell assay, the reason for using the same concentration of A485 in both the upper and lower chambers should be clarified.

Regarding the improvement of result analysis:

It is mentioned in Figure 1 that "ac-EP300 is significantly increased in 2/4 tumor tissues". Specific statistical analysis results, including sample size and P-value, should be supplemented to more strongly support the association between EP300 activation and the malignant progression of bladder cancer.

In the RNAseq analysis, for the 229 commonly downregulated genes, their specific functional classifications and direct evidence of association with SNAI2-related pathways should be supplemented, such as whether there are target genes of SNAI2.

Regarding the deepening of the discussion section:

The discussion mentions that EP300 may have oncogenic or tumor-suppressive effects in different cancers. Combined with the results of this study, it is necessary to further elaborate on the specificity and potential risks of targeting EP300 as a therapeutic strategy in bladder cancer.

The basis for selecting the concentration of A485 (10μM) in this study should be supplemented, such as whether it has been verified by pre-experiments, as well as references regarding the possible safety and effectiveness of this concentration in vivo.

Regarding data availability and ethical statements:

The data availability section mentions that all data are fully available without restriction. Specific access methods, such as database names and accession numbers, should be supplemented.

In the ethical approval section, it is necessary to clarify whether informed consent was obtained from the participants during the collection of human samples and the form of consent (written or oral).

Please revise and improve the manuscript according to the above comments. If the responses are appropriate, this manuscript is suitable for publication in PLOS ONE.

Reviewer #2: This study investigates the role of EP300 in bladder cancer migration. It confirms the positive association between EP300 and bladder malignancy. The study observed A485, which is a small-molecule inhibitor of EP300, significantly inhibited the migration of multiple bladder cancer cell lines (SW780, T24 ,RT4, 5637) both in scratch wound healing and Transwell assays, and disrupted organoid migration in 3D culture. Using bulk RNA-seq analysis and western blotting the research found that A485 downregulates SNAI2-related signaling pathways and reduce the expression of SNAI2 and the H3K27ac level. Overexpression of SNAI2 enhances the bladder cancer cell migration, and EP300 can promote bladder cancer cell migration via SNAI2, and targeting EP300 could be a potential therapeutic strategy.

1、Lack of in vivo validation for the effects of EP300 inhibition. Effects the translational medical value of the conclusion.

2、The results only used 4 matched pairs of bladder cancer and adjacent tissues. Is the sample size too small?

3、The discussion says that the EP300 has two opposite effects, but the study only explores the EP300’s role of promoting migratory, and don’t delve into the possibility of EP300 suppressing the bladder cancer, nor does it analyze the influence of these opposite effects for EP300-targeted therapeutic strategies.

4、The discussion only repeats the results of the study, lacks an analysis of the limitation of the study.

5、Figure5, only inhibition of EP300 via A485 alone led to a decrease in SNAI2 expression was observed. Lack the direct evidence to demonstrate that EP300 binds to the promoter or enhancer region of SNAI2. It is recommended to supplement the study with experiments(such as ChIP-qPCR and luciferase reporter assay) to validate the regulatory relationship.

**********

6. PLOS authors have the option to publish the peer review history of their article (what does this mean?). If published, this will include your full peer review and any attached files.

If you choose “no”, your identity will remain anonymous but your review may still be made public.

**Do you want your identity to be public for this peer review?** For information about this choice, including consent withdrawal, please see our Privacy Policy.

Reviewer #1: No

Reviewer #2: No

**********

[NOTE: If reviewer comments were submitted as an attachment file, they will be attached to this email and accessible via the submission site. Please log into your account, locate the manuscript record, and check for the action link "View Attachments". If this link does not appear, there are no attachment files.]

While revising your submission, please upload your figure files to the Preflight Analysis and Conversion Engine (PACE) digital diagnostic tool, https://pacev2.apexcovantage.com/. PACE helps ensure that figures meet PLOS requirements. To use PACE, you must first register as a user. Registration is free. Then, login and navigate to the UPLOAD tab, where you will find detailed instructions on how to use the tool. If you encounter any issues or have any questions when using PACE, please email PLOS at figures@plos.org. Please note that Supporting Information files do not need this step.

# Author comment: Author response to Decision Letter 1

**DOI:** 10.1371/journal.pone.0347209.r002

12 Nov 2025

Dear Editor Chien-Feng Li,

Thank you for your kind help and effort on our manuscripts. We have addressed all question from reviewers, please process the Revised manuscripts. Thank you.

Chunming Guo

Attachment Submitted filename: Response letter_Plos One .docx

# Peer review: Decision Letter 1

**DOI:** 10.1371/journal.pone.0347209.r003

**Authors:** Chien-Feng Li

16 Dec 2025

PONE-D-25-21042R1

EP300 promotes bladder cancer cell migration through SNAI2

PLOS One

Dear Dr. Guo,

Thank you for submitting your manuscript to PLOS ONE. After careful consideration, we feel that it has merit but does not fully meet PLOS ONE’s publication criteria as it currently stands. Therefore, we invite you to submit a revised version of the manuscript that addresses the points raised during the review process.

- **Please revise your work as per the reviewers' comments.**

-->Please submit your revised manuscript by Jan 30 2026 11:59PM. If you will need more time than this to complete your revisions, please reply to this message or contact the journal office at plosone@plos.org. When you're ready to submit your revision, log on to https://www.editorialmanager.com/pone/ and select the 'Submissions Needing Revision' folder to locate your manuscript file.

Please include the following items when submitting your revised manuscript:

- A rebuttal letter that responds to each point raised by the academic editor and reviewer(s). You should upload this letter as a separate file labeled 'Response to Reviewers'.
- A marked-up copy of your manuscript that highlights changes made to the original version. You should upload this as a separate file labeled 'Revised Manuscript with Track Changes'.
- An unmarked version of your revised paper without tracked changes. You should upload this as a separate file labeled 'Manuscript'.

-->If you would like to make changes to your financial disclosure, please include your updated statement in your cover letter. Guidelines for resubmitting your figure files are available below the reviewer comments at the end of this letter.

If applicable, we recommend that you deposit your laboratory protocols in protocols.io to enhance the reproducibility of your results. Protocols.io assigns your protocol its own identifier (DOI) so that it can be cited independently in the future. For instructions see: https://journals.plos.org/plosone/s/submission-guidelines#loc-laboratory-protocols. Additionally, PLOS ONE offers an option for publishing peer-reviewed Lab Protocol articles, which describe protocols hosted on protocols.io. Read more information on sharing protocols at https://plos.org/protocols?utm_medium=editorial-email&utm_source=authorletters&utm_campaign=protocols.

We look forward to receiving your revised manuscript.

Kind regards,

Chien-Feng Li, M.D., Ph.D.

Academic Editor

PLOS One

**Journal Requirements:**

1. If the reviewer comments include a recommendation to cite specific previously published works, please review and evaluate these publications to determine whether they are relevant and should be cited. There is no requirement to cite these works unless the editor has indicated otherwise.

2. Please review your reference list to ensure that it is complete and correct. If you have cited papers that have been retracted, please include the rationale for doing so in the manuscript text, or remove these references and replace them with relevant current references. Any changes to the reference list should be mentioned in the rebuttal letter that accompanies your revised manuscript. If you need to cite a retracted article, indicate the article’s retracted status in the References list and also include a citation and full reference for the retraction notice.

[Note: HTML markup is below. Please do not edit.]

Reviewers' comments:

Reviewer's Responses to Questions

**Comments to the Author**

1. If the authors have adequately addressed your comments raised in a previous round of review and you feel that this manuscript is now acceptable for publication, you may indicate that here to bypass the “Comments to the Author” section, enter your conflict of interest statement in the “Confidential to Editor” section, and submit your "Accept" recommendation.

Reviewer #1: All comments have been addressed

Reviewer #2: All comments have been addressed

**********

2. Is the manuscript technically sound, and do the data support the conclusions?

The manuscript must describe a technically sound piece of scientific research with data that supports the conclusions. Experiments must have been conducted rigorously, with appropriate controls, replication, and sample sizes. The conclusions must be drawn appropriately based on the data presented.

Reviewer #1: Yes

Reviewer #2: Yes

**********

3. Has the statistical analysis been performed appropriately and rigorously?

Reviewer #1: Yes

Reviewer #2: Yes

**********

4. Have the authors made all data underlying the findings in their manuscript fully available?

The PLOS Data policy requires authors to make all data underlying the findings described in their manuscript fully available without restriction, with rare exception (please refer to the Data Availability Statement in the manuscript PDF file). The data should be provided as part of the manuscript or its supporting information, or deposited to a public repository. For example, in addition to summary statistics, the data points behind means, medians and variance measures should be available. If there are restrictions on publicly sharing data—e.g. participant privacy or use of data from a third party—those must be specified.

Reviewer #1: Yes

Reviewer #2: No

**********

5. Is the manuscript presented in an intelligible fashion and written in standard English?

PLOS ONE does not copyedit accepted manuscripts, so the language in submitted articles must be clear, correct, and unambiguous. Any typographical or grammatical errors should be corrected at revision, so please note any specific errors here.

Reviewer #1: Yes

Reviewer #2: Yes

**********

6. Review Comments to the Author

Please use the space provided to explain your answers to the questions above. You may also include additional comments for the author, including concerns about dual publication, research ethics, or publication ethics. (Please upload your review as an attachment if it exceeds 20,000 characters)

**Reviewer #1:** Recommendations to Strengthen the Evidence for EP300-SNAI2 Mediated Migration in Bladder Cancer

The manuscript by Zhang et al. investigates the role of EP300 in promoting bladder cancer cell migration via the transcription factor SNAI2. The study employs a multi-faceted approach, including IHC on patient samples, in vitro 2D/3D migration assays, in vivo mouse models, RNA-seq, and molecular biology techniques. The central finding—that the EP300 inhibitor A485 suppresses migration by downregulating SNAI2—is novel and of potential interest to the field of bladder cancer therapeutics. However, the manuscript in its current form requires revisions to strengthen the mechanistic claims, improve data rigor, and enhance clarity before it can be considered for publication.

Major Concerns:

1. Insufficient Mechanistic Evidence

How can you demonstrate that EP300 directly regulates SNAI2, rather than through indirect pathways? The use of inhibitors and H3K27ac CUT&Tag alone is insufficient to prove direct regulation. Can you provide EP300-specific ChIP experimental data? Furthermore, can SNAI2 overexpression fully reverse the inhibitory effect of A485 on migration, thereby proving it is the key downstream effector of EP300?

2. Over-reliance on a Single Experimental Approach

The entire study heavily depends on a single inhibitor, A485. To exclude potential off-target effects, can you provide genetic evidence (e.g., EP300 knockdown or knockout) demonstrating that the observed phenotypes (inhibited migration and downregulated SNAI2) are consistent with pharmacological inhibition?

3. Inadequate Support for Conclusions

The manuscript states that A485 only shows a "trend" in vivo without statistical significance. Under these circumstances, why is this still presented as key supporting evidence? Please provide detailed sample sizes (n values) and statistical methods, and discuss the impact of this non-significant result on your conclusions.

4. Lack of Essential Control Experiments

How have you verified that the inhibition of migration by A485 is not simply due to cytotoxicity or proliferation suppression? Please provide cell viability/proliferation data under the exact same conditions used in the migration assays as supplementary information.

5. Data Presentation Issues

Please comprehensively check and correct all figure citations throughout the manuscript.

**Reviewer #2:** Your study elegantly identifies the EP300-SNAI2 axis in bladder cancer cell migration, with solid in vitro, 3D culture, and in vivo evidence. The revised data, including expanded clinical samples (12 pairs) and GSA-deposited RNA-seq/CUT&Tag data (PRJCA038992), significantly strengthens the work. However, two key issues require attention. First, per PLOS ONE’s mandates, uncropped, unadjusted blot/gel images (e.g., Western blot for SNAI2 and H3K27ac) remain unpublished—please deposit these in the supplementary materials or a public repository. Second, the in vivo BBN model shows a trend of reduced invasion but lacks statistical significance, possibly due to small sample size (n=4 per group); expanding the cohort or adding supplementary functional assays (e.g., metastasis tracking) would reinforce translational relevance.

**********

7. PLOS authors have the option to publish the peer review history of their article (what does this mean?). If published, this will include your full peer review and any attached files.

If you choose “no”, your identity will remain anonymous but your review may still be made public.

**Do you want your identity to be public for this peer review?** For information about this choice, including consent withdrawal, please see our Privacy Policy.

Reviewer #1: No

Reviewer #2: No

**********

[NOTE: If reviewer comments were submitted as an attachment file, they will be attached to this email and accessible via the submission site. Please log into your account, locate the manuscript record, and check for the action link "View Attachments". If this link does not appear, there are no attachment files.]

To ensure your figures meet our technical requirements, please review our figure guidelines: https://journals.plos.org/plosone/s/figures

You may also use PLOS’s free figure tool, NAAS, to help you prepare publication quality figures: https://journals.plos.org/plosone/s/figures#loc-tools-for-figure-preparation.

NAAS will assess whether your figures meet our technical requirements by comparing each figure against our figure specifications.

# Author comment: Author response to Decision Letter 2

**DOI:** 10.1371/journal.pone.0347209.r004

29 Jan 2026

We are deeply grateful to the reviewers for their insightful and constructive feedback on our manuscript entitled "EP300 promotes bladder cancer cell migration through SNAI2." The critiques have been invaluable in helping us strengthen the evidence and clarify the presentation of our study. Below, we provide a point-by-point response to each comment. All planned revisions and additions to the manuscript are detailed herein, and corresponding changes will be clearly highlighted in the revised manuscript.

Attachment Submitted filename: +Response letter_Plos One-R2.docx

# Editor report: Decision Letter 2

**DOI:** 10.1371/journal.pone.0347209.r005

**Authors:** Habib Boukerche

30 Mar 2026

EP300 promotes bladder cancer cell migration through SNAI2

PONE-D-25-21042R2

Dear Dr. Chunming Guo,

We’re pleased to inform you that your manuscript has been judged scientifically suitable for publication and will be formally accepted for publication once it meets all outstanding technical requirements.

Within one week, you’ll receive an e-mail detailing the required amendments. When these have been addressed, you’ll receive a formal acceptance letter and your manuscript will be scheduled for publication.

An invoice will be generated when your article is formally accepted. Please note, if your institution has a publishing partnership with PLOS and your article meets the relevant criteria, all or part of your publication costs will be covered. Please make sure your user information is up-to-date by logging into Editorial Manager at Editorial Manager® and clicking the ‘Update My Information' link at the top of the page. For questions related to billing, please contact billing support.

If your institution or institutions have a press office, please notify them about your upcoming paper to help maximize its impact. If they’ll be preparing press materials, please inform our press team as soon as possible -- no later than 48 hours after receiving the formal acceptance. Your manuscript will remain under strict press embargo until 2 pm Eastern Time on the date of publication. For more information, please contact onepress@plos.org.

Kind regards,

Dr H. Boukerche, PhD

Academic Editor

PLOS One

Additional Editor Comments (optional):

Reviewers' comments:

# Editor report: Acceptance letter

**DOI:** 10.1371/journal.pone.0347209.r006

**Authors:** Habib Boukerche

PONE-D-25-21042R2

PLOS One

Dear Dr. Guo,

I'm pleased to inform you that your manuscript has been deemed suitable for publication in PLOS One. Congratulations! Your manuscript is now being handed over to our production team.

At this stage, our production department will prepare your paper for publication. This includes ensuring the following:

* All references, tables, and figures are properly cited

* All relevant supporting information is included in the manuscript submission,

* There are no issues that prevent the paper from being properly typeset

You will receive further instructions from the production team, including instructions on how to review your proof when it is ready. Please keep in mind that we are working through a large volume of accepted articles, so please give us a few days to review your paper and let you know the next and final steps.

Lastly, if your institution or institutions have a press office, please let them know about your upcoming paper now to help maximize its impact. If they'll be preparing press materials, please inform our press team within the next 48 hours. Your manuscript will remain under strict press embargo until 2 pm Eastern Time on the date of publication. For more information, please contact onepress@plos.org.

You will receive an invoice from PLOS for your publication fee after your manuscript has reached the completed accept phase. If you receive an email requesting payment before acceptance or for any other service, this may be a phishing scheme. Learn how to identify phishing emails and protect your accounts at https://explore.plos.org/phishing.

If we can help with anything else, please email us at customercare@plos.org.

Thank you for submitting your work to PLOS ONE and supporting open access.

Kind regards,

PLOS ONE Editorial Office Staff

on behalf of

Dr Habib Boukerche

Academic Editor

PLOS One
